# Supplementary material for: The RIX domain defines a class of polymorphic T6SS effectors and secreted adaptors
Source: Nat Commun. 2023 Aug 17;14:4983. doi: 10.1038/s41467-023-40659-2 (PMC10435454; doi:10.1038/s41467-023-40659-2)
Supplement: Supplementary file 1 — Supplementary Information [file 41467_2023_40659_MOESM1_ESM.pdf]

Supplementary Information

The RIX domain defines a class of polymorphic T6SS effectors and secreted adaptors

- **Figures S1-S9**
- **Tables S1-S3**
- **References**

Supplementary Figures

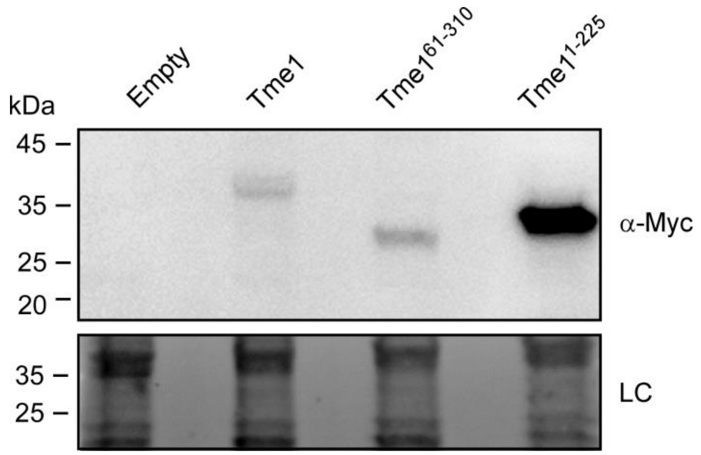

Fig. S1. Tme1 truncations are expressed in *E. coli*. Expression of the indicated C-terminal Myc-His tagged Tme1 forms from arabinose-inducible pPER5-based plasmids in *E. coli* BL21 (DE3). Proteins were detected by immunoblotting using specific α-Myc antibodies. Loading control (LC) is shown for total protein lysates. The experiment was repeated five independent times with similar results. Results from a representative experiment are shown. Source data are provided as a Source Data file.

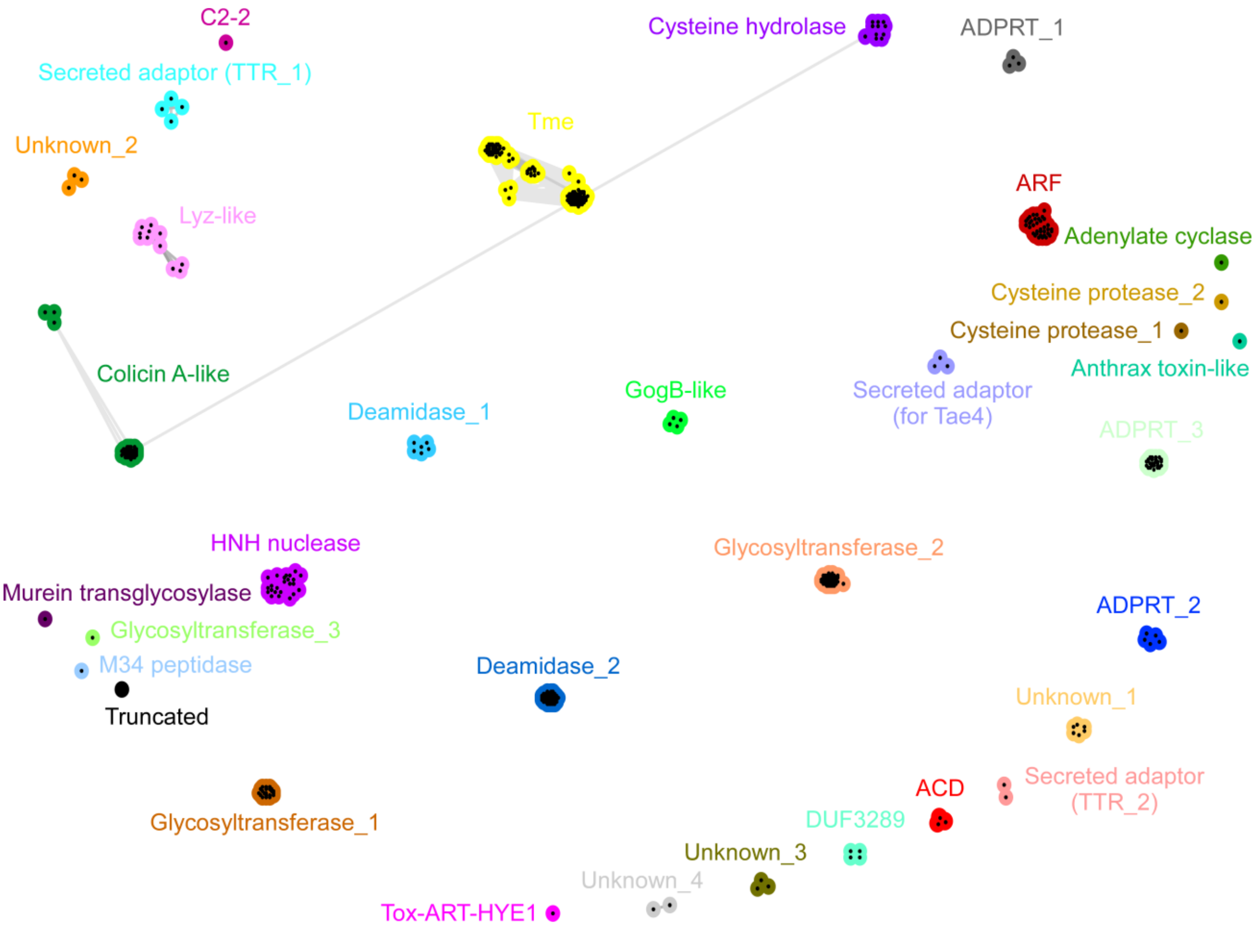

Fig. S2. RIX domain C-terminal extensions cluster into 33 distinct families. C-termini of RIX domain-containing proteins clustered in two dimensions. Clustering was performed based on all-against-all sequence similarity, with nodes representing unique sequences and connecting lines representing the distances between sequences. The predicted activities or domains identified in each cluster are denoted. Lyz, lysozyme; ADPRT, ADP ribosyl transferase; ARF, ADP ribosylation factor; ACD, Actin cross-linking; TTR, Transthyretin-like.

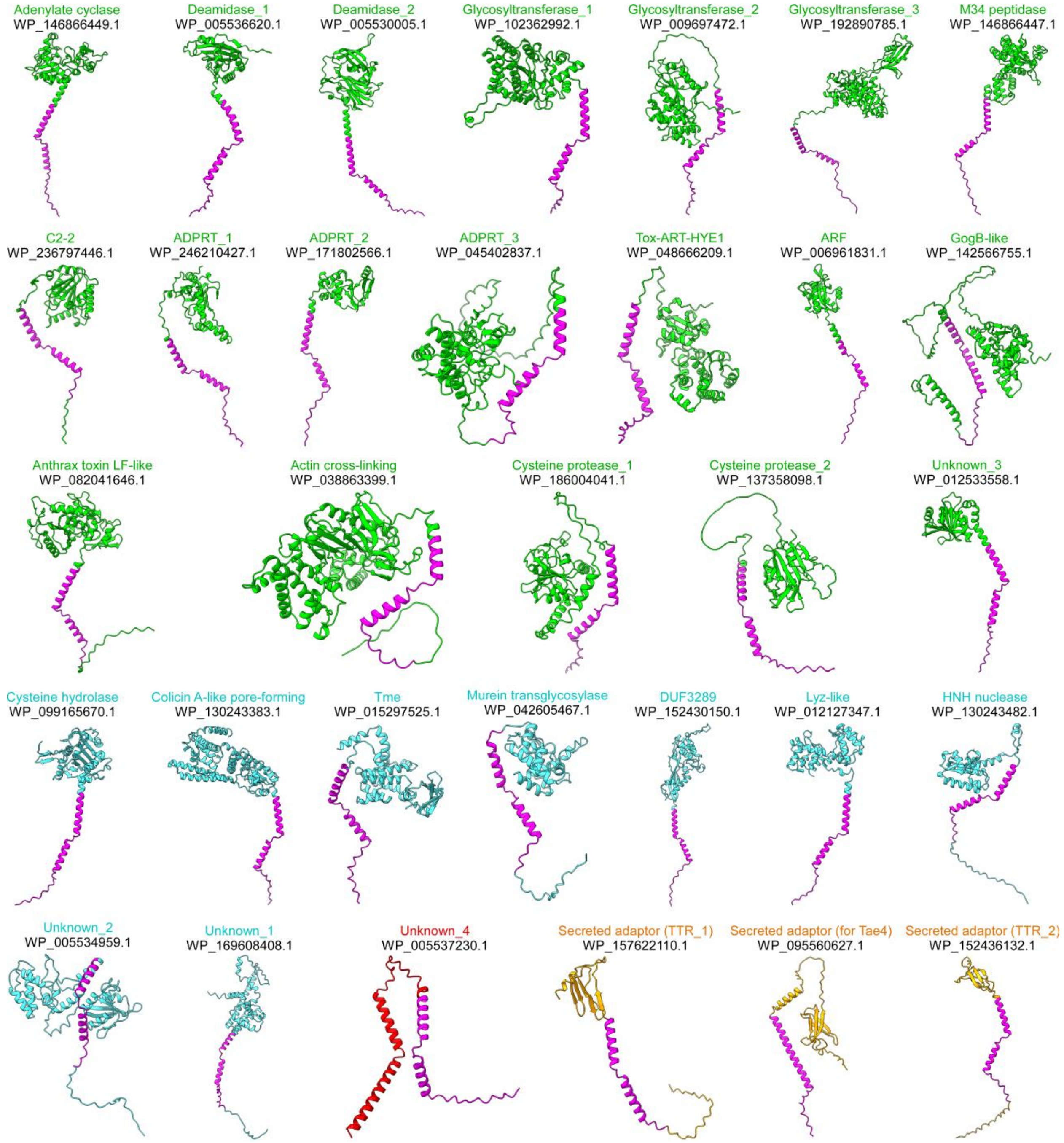

Fig. S3. AlphaFold2 structure predictions of RIX cluster representatives. The structures of a representative protein from 32 RIX C-termini clusters (excluding the “truncated” cluster) were predicted using AlphaFold2. The protein accession number and the predicted activity or domain are denoted above. Regions corresponding to RIX are denoted in magenta; proteins with predicted anti-eukaryotic activities are denoted in green; proteins with predicted antibacterial activities are denoted in cyan; proteins with predicted secreted adaptor activities are denoted in orange; C-terminal extensions with an unknown function are denoted in red. Lyz, lysozyme; ADPRT, ADP ribosyl transferase; ARF, ADP ribosylation factor; TTR, Transthyretin-like.

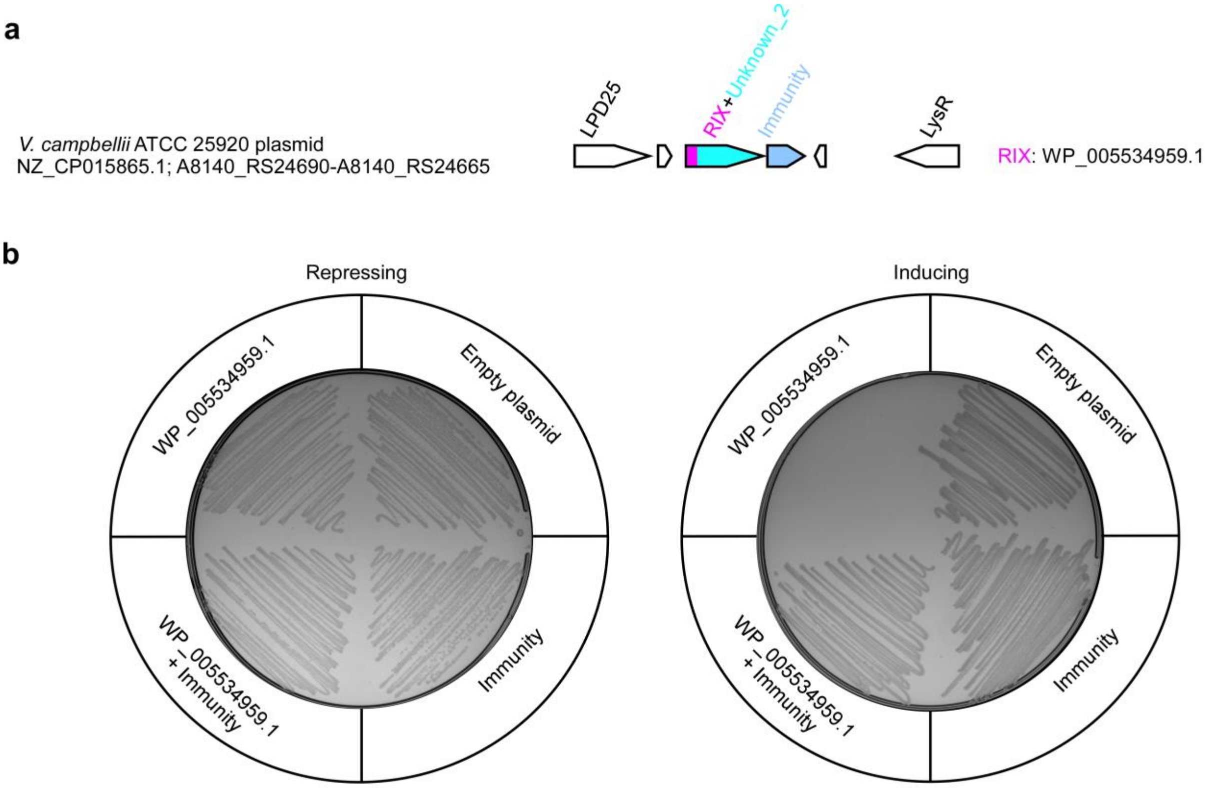

Fig. S4. The RIX domain-containing WP_005534959.1 is an antibacterial toxin. (a) The genomic neighborhood of the gene encoding WP_005534959.1 in *V. campbellii* ATCC 25920. The strain name, the GenBank accession number, and the locus tags are provided. Genes are denoted by arrows indicating the direction of transcription. The names of encoded proteins or domains are denoted above. **(b)** Toxicity of WP_005534959.1 expressed from pBAD33.1^{F}-based arabinose-inducible plasmids in *E. coli* BL21 (DE3), with or without its downstream-encoded predicted immunity protein. Bacteria were streaked onto repressing (4% wt/vol glucose) or inducing (0.05% wt/vol arabinose) agar plates. The experiment was repeated four independent times with similar results. Results from a representative experiment are shown.

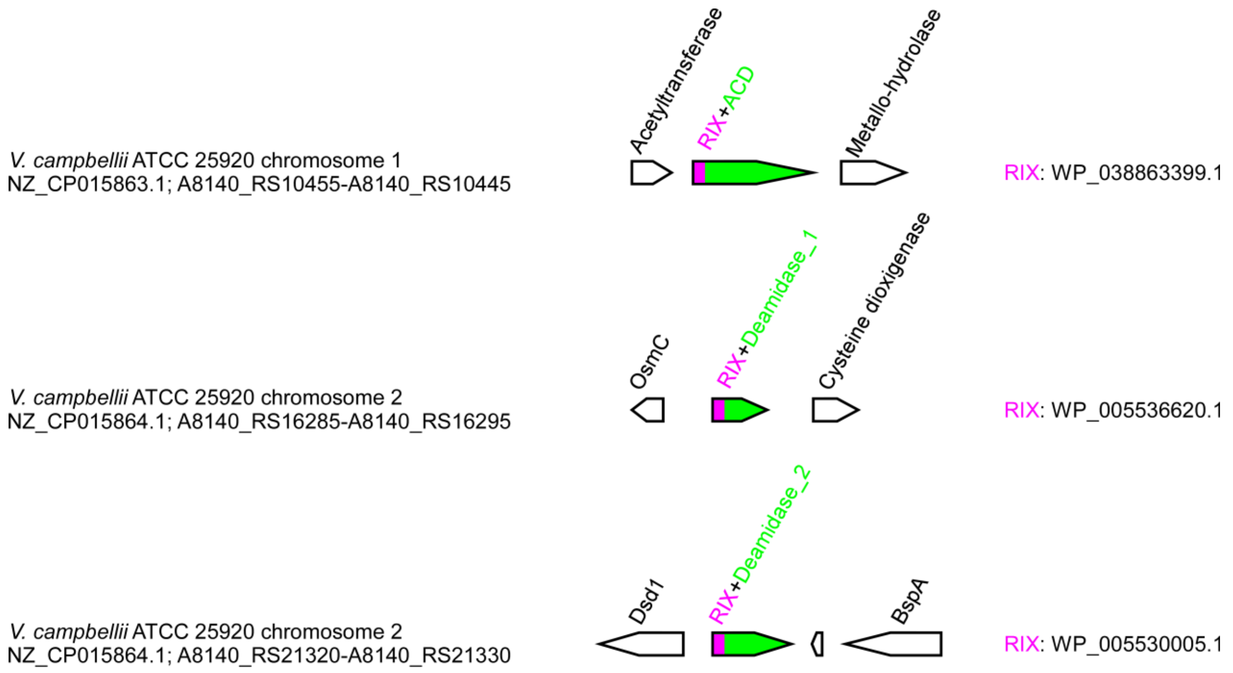

Fig. S5. Three genes encoding RIX domain-containing proteins with predicted anti-eukaryotic activities in *V. campbellii*. The genomic neighborhood of the genes encoding the indicated RIX domain-containing proteins in *V. campbellii* ATCC 25920. The strain name, the GenBank accession number, and the locus tags are provided. Genes are denoted by arrows indicating the direction of transcription. The names of encoded proteins or domains are denoted above. ACD, Actin cross-linking.

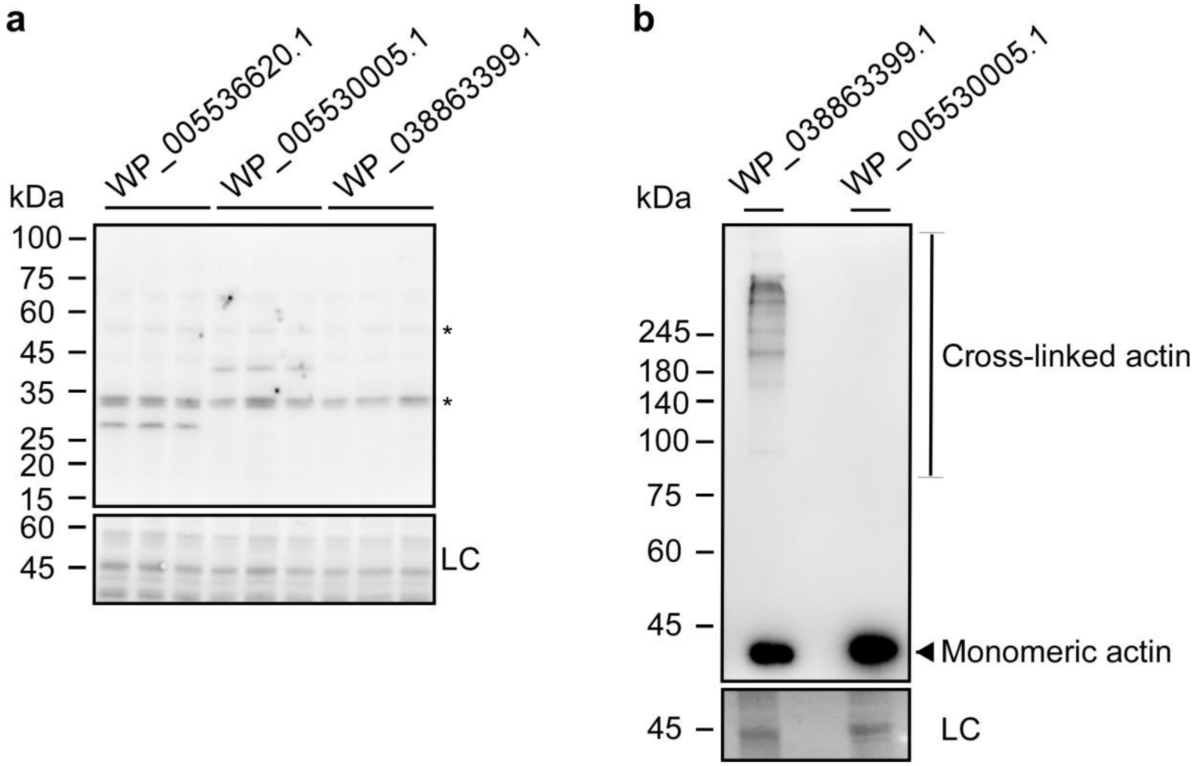

Fig. S6. Expression of predicted anti-eukaryotic RIX domain-containing proteins in yeast. (a) Expression of the indicated C-terminal Myc-tagged proteins from galactose-inducible plasmids in yeast. WP_005536620.1 (deamidase_1) is predicted to be a 28 kDa protein, WP_005530005.1 (deamidase_2) is predicted to be a 39 kDa protein, and WP_038863399.1 (actin cross-linking) is predicted to be a 58 kDa protein. Lysates from three colonies for each expressed protein were used. Proteins were detected by immunoblotting using specific α-Myc antibodies. Loading control (LC) is shown for total protein lysates. Asterisks denote non-specific bands detected by the α-Myc antibody. **(b)** Actin forms found in lysates of yeast expressing the indicated proteins from a galactose-inducible plasmid were detected by immunoblotting using α-β-actin antibodies. Loading control (LC) is shown for total protein lysates. WP_005530005.1 (deamidase_2) was used as a non-actin cross-linking negative control. The experiments were repeated three independent times with similar results. Results from representative experiments are shown. Source data are provided as a Source Data file.

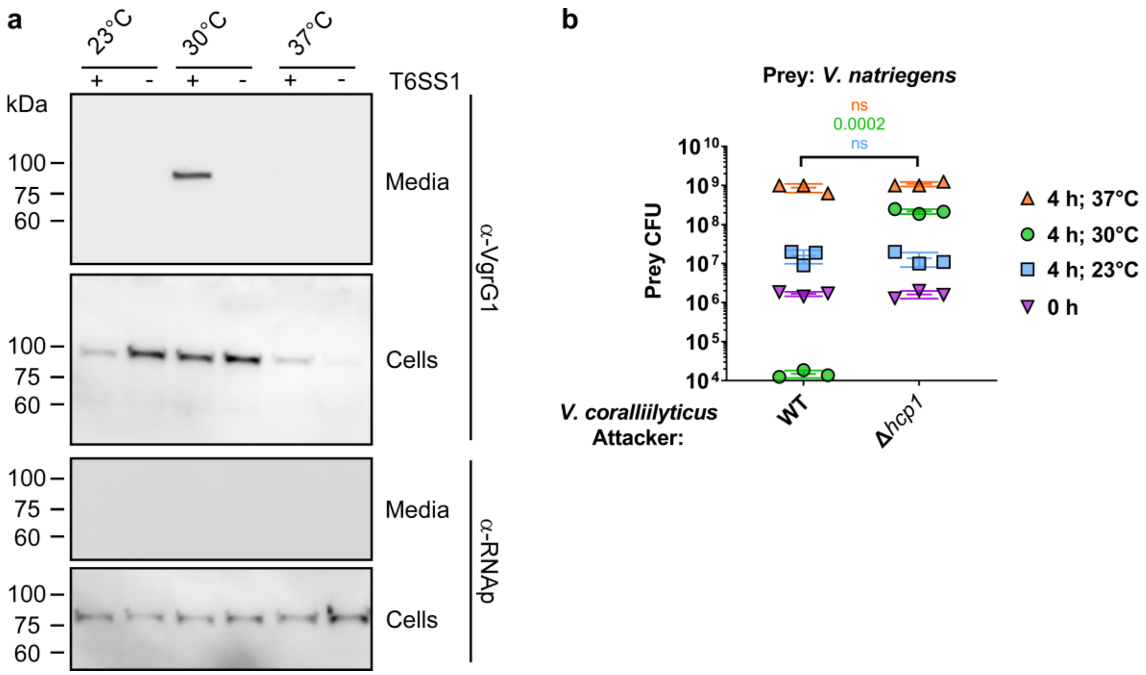

Fig. S7. *V. coralliilyticus* T6SS1 mediates antibacterial toxicity under warm, marine-like conditions. (a) Expression (cells) and secretion (media) of VgrG1 from *V. coralliilyticus* BAA-450 wild type (T6SS1$^+$) or Δ*hcp1* (T6SS1$^-$) strains. Samples were grown in MLB media for 4 h at the indicated temperature. RNA polymerase sigma 70 (RNAp) was used as loading and lysis control. The experiment was repeated three independent times with similar results. Results from a representative experiment are shown. **(b)** Viability counts of *V. natriegens* prey before (0 h) and after (4 h) co-incubation with the indicated *V. coralliilyticus* BAA-450 attacker at the indicated temperature. The statistical significance between samples at the 4 h timepoint (color coded to match the relevant samples) was calculated using an unpaired, two-tailed Student's *t*-test; ns, no significant difference ($p>0.05$). Data are shown as the mean ± SD; n = 3 biological samples. The experiment was repeated three independent times with similar results. Results from a representative experiment are shown. WT, wild type. Source data are provided as a Source Data file.

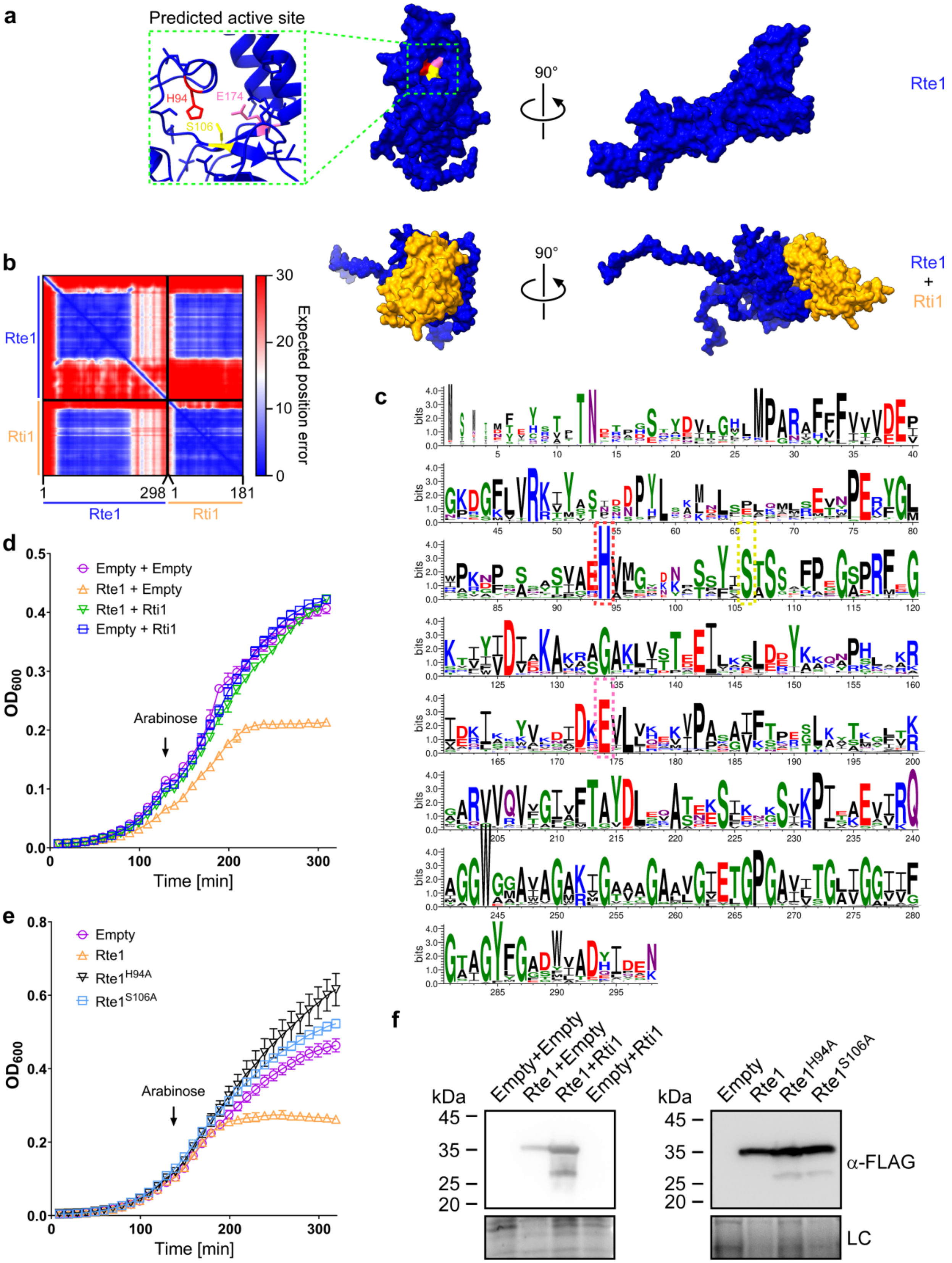

a
Predicted active site
H94
E174
S106
90°
Rte1
b
Rte1
Rti1
30
20
10
0
Expected position error
1
298
1
181
Rte1
Rti1
90°
Rte1
+
Rti1
c
d
Empty + Empty
Rte1 + Empty
Rte1 + Rti1
Empty + Rti1
Arabinose
OD600
Time [min]
e
Empty
Rte1
Rte1H94A
Rte1S106A
Arabinose
OD600
Time [min]
f
Empty+Empty
Rte1+Empty
Rte1+Rti1
Empty+Rti1
kDa
45
35
25
20
Empty
Rte1
Rte1H94A
Rte1S106A
α-FLAG
LC

Fig. S8. Rte1 and Rti1 are an antibacterial effector and immunity pair. (a) The structure of Rte1, either alone (top) or in complex with Rti1 (bottom) was predicted using AlphaFold2. Predicted active site residues are shown inside the green dashed rectangle. **(b)** The predicted aligned error of the complex shown in (a). A low predicted aligned error value indicates that the predicted relative position and orientation of two residues is well defined. **(c)** A conservation logo of Rte1 is illustrated using WebLogo3, based on multiple sequence alignment of Rte1 homologs. The position numbers correspond to the amino acids in Rte1. Predicted active site residues are denoted by a dashed rectangle, and color coded to match the residues shown in (a). **(d-e)** Growth of *E. coli* BL21 (DE3) containing plasmids for the arabinose-inducible expression of the indicated proteins. An arrow denotes the timepoint at which arabinose (0.05% wt/vol) was added to the media. In (d), Rte1 and Rti1 were expressed from separate plasmids (pBAD33.1^{F} and pBAD^{K}/Myc-His-based, respectively. Data are shown as the mean ± SD; n = 4 biological samples. The experiments were repeated three independent times with similar results. Results from representative experiments are shown. **(f)** Expression of the indicated C-terminal FLAG tagged Rte1 forms from arabinose-inducible plasmids in *E. coli* BL21 (DE3) strains used in (d) and (e). Proteins were detected by immunoblotting using specific α-FLAG antibodies. Loading control (LC) is shown for total protein lysates. The experiment was repeated three independent times with similar results. Results from a representative experiment are shown. Source data are provided as a Source Data file.

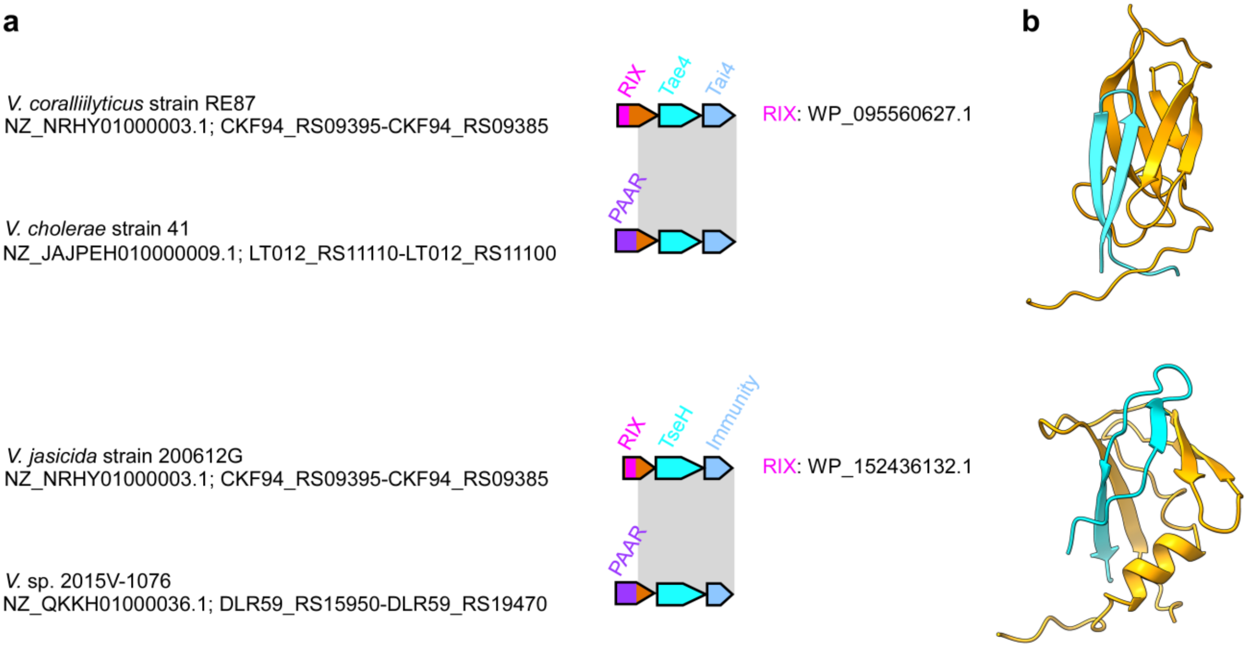

Fig. S9. RIX domain-containing proteins can be secreted adaptors for cargo effector. **(a)** The gene structure of the operons encoding WP_095560627.1 in *V. coralliilyticus* RE87 and WP_152436132.1 in *V. jasicida* 200612G. Operons encoding a module homologous to the C-terminal extension of the RIX domain-containing protein and the downstream-encoded effector and immunity pair are shown below; a gray rectangle denotes the region of homology. The strain names, the GenBank accession numbers, and the locus tags are provided. Genes are denoted by arrows indicating the direction of transcription. The names of encoded proteins or domains are denoted above. **(b)** Interactions between the RIX-containing proteins and their downstream-encoded effector were predicted by AlphaFold2. The interaction interfaces, corresponding to amino acids 102-185 of WP_095560627.1 (orange) and 1-21 of Tae4 (cyan) (top), and amino acids 76-134 of WP_152436132.1 (orange) and 1-24 of TseH (cyan) (bottom), are shown.

Supplementary Tables

Table S1. A list of bacterial strains used in this study.

Strain name	Genotype	Comments	Source
Vibrio parahaemolyticus BB22OP	Wild type	Used for generating deletion strains	Obtained from Kim Orth; [1]
Vibrio parahaemolyticus BB22OP Δ*hns*/Δ*tme1*	Δ*vpbb_rs05425*/ Δ*vpbb_rs15030*	BB22OP derivative containing in-frame deletions of *hns* and *tme1*. Used for secretion assays	[2]
Vibrio parahaemolyticus BB22OP Δ*hns*/Δ*tme1*/Δ*hcp1*	Δ*vpbb_rs05425*/ Δ*vpbb_rs15030*/ Δ*vpbb_rs06665*	BB22OP derivative containing in-frame deletions of *hns, tme1,* and *hcp1*. Used for secretion assays	[2]
Vibrio parahaemolyticus RIMD 2210633	Wild type	Used for generating deletion strains	Obtained from Kim Orth
Vibrio parahaemolyticus RIMD 2210633 Δ*hns*/Δ*tdhAS*/ Δ*vp_rs06130*/ Δ*vp_rs06745*/ *vp_rs06875*^{AAA}<br><br>(Surrogate attacker)	Δ*vp_rs05510*/Δ*tdhAS*/ Δ*vp_rs06130*/ Δ*vp_rs06745*/ *vp_rs06875*^{AAA}	RIMD 2210633 derivative with a constitutively active, effectorless T6SS1. Used as a surrogate platform in competition assays and in secretion assays	[3]
Vibrio parahaemolyticus RIMD 2210633 Δ*hns*/Δ*tdhAS*/ Δ*vp_rs06130*/ Δ*vp_rs06745*/ *vp_rs06875*^{AAA}/Δ*hcp1*	Surrogate attacker Δ*hcp1*	Surrogate attacker derivative with an inactive T6SS1. Used as a surrogate platform in competition assays and in secretion assays	[3]
Vibrio natriegens ATCC 14048	Wild type	Used as prey in competition assays	ATCC collection

Vibrio coralliilyticus ATCC BAA-450	Wild type	Used for generating deletion strains, as the attacker in competition assays, and in secretion assays	ATCC collection
Vibrio coralliilyticus ATCC BAA-450 Δ*hcp1*	Δ*vic_rs16330*	ATCC BAA-450 derivative containing an in-frame deletion of *vic_rs16330*; used as the attacker in competition assays, and in secretion assays	[3]
Vibrio campbellii ATCC 25920	Wild type	Used for cloning	ATCC collection
Escherichia coli DH5α (λ pir)	K-12 derivative laboratory strain containing λ pir	Used for plasmid maintenance and cloning	Obtained from Eric V. Stabb
Escherichia coli BL21 (DE3)	Laboratory strain	Used for protein expression and toxicity assays	Obtained from Kim Orth

Table S2. A list of plasmids used in this study.

Plasmid name	Description	Purpose	Source
pBAD^{K}/Myc-His	pBR322 ori-containing plasmid harboring a Kan^{R} cassette, *araC*, and an MCS following a P*bad* promoter	Used for arabinose-inducible expression	[4]
pRti1^{M}	pBAD^{K}/Myc-His plasmid containing the CDS of WP_050778602.1 from *V. coralliilyticus* ATCC BAA-450 in frame with a C-terminal Myc-His tag	Used for the arabinose-inducible expression of WP_050778602.1 in *E. coli* and *V. parahaemolyticus*	This study
pPER5	pBAD^{K}/Myc-His with a PelB signal peptide inserted at the 5' end of the MCS	Used for the arabinose-inducible expression of proteins targeted to the periplasm in *E. coli*	[5]
pTme1^{peri}	pPER5 plasmid containing the CDS	Used for the arabinose-inducible expression of	[2]

	of Tme1 (WP_015297525.1) from *V. parahaemolyticus* BB22OP in frame with an N-terminal PelB signal peptide and the C-terminal Myc-His tag	Tme1 protein targeted to the periplasm in *E. coli*	
pTme1$^{61\text{-}310/peri}$	pPER5 plasmid containing the CDS of Tme1$^{61\text{-}310}$ (truncation of the first 60 amino acids of Tme1) in frame with an N-terminal PelB signal peptide and a C-terminal Myc-His tag	Used for the arabinose-inducible expression of Tme1$^{61\text{-}310}$ protein targeted to the periplasm in *E. coli*	This study
pTme1$^{1\text{-}225/peri}$	pPER5 plasmid containing the CDS of Tme1$^{1\text{-}225}$ (truncation of amino acids 226-310 of Tme1) in frame with an N-terminal PelB signal peptide and a C-terminal Myc-His tag	Used for the arabinose-inducible expression of Tme1$^{1\text{-}225}$ protein targeted to the periplasm in *E. coli*	This study
pBAD33.1^{F}	pBAD33.1 with a FLAG tag inserted at the 3' end of the MCS	Used for the arabinose-inducible expression of proteins	[2]
pTme1^{M}	pBAD33.1^{F} plasmid amplified without the FLAG tag, containing CDS of Tme1 in frame with the C-terminal Myc-His tag	Used for the arabinose-inducible expression of proteins in *V. parahaemolyticus* BB22OP	This study
pTme1$^{61\text{-}310M}$	pBAD33.1^{F} plasmid amplified without the FLAG tag, containing CDS of Tme1$^{61\text{-}310}$ (truncation of the first 60 amino acids of Tme1) in frame with the C-terminal Myc-His tag	Used for the arabinose-inducible expression of proteins in *V. parahaemolyticus* BB22OP	This study

sfGFP-N1	Eukaryotic expression plasmid encoding superfolder GFP (sfGFP)	Used as a template to amplify *sfgfp*	Addgene
pTme1$^{1-60}$-sfGFP	pBAD33.1^{F} plasmid containing CDS for Tme1$^{1-60}$ (the first 60 amino acids of Tme1) fused to sfGFP, in frame with the C-terminal FLAG tag of the plasmid	Used for the arabinose-inducible expression of proteins in *V. parahaemolyticus* BB22OP	This study
psfGFP	pBAD33.1^{F} plasmid containing CDS of sfGFP in frame with the C-terminal FLAG tag of the plasmid	Used for the arabinose-inducible expression of proteins in *V. parahaemolyticus* BB22OP	[6]
pWP_005536620.1	pBAD33.1^{F} plasmid containing CDS of WP_005536620.1 from *V. campbellii* ATCC 25920 in frame with the C-terminal FLAG tag of the plasmid	Used for the arabinose-inducible expression of proteins in *V. parahaemolyticus*	This study
pWP_005536620.1$^{64-241}$	pBAD33.1^{F} plasmid containing CDS of WP_005536620.1$^{64-241}$ (truncation of the first 63 amino acids of WP_005536620.1) in frame with the C-terminal FLAG tag of the plasmid	Used for the arabinose-inducible expression of proteins in *V. parahaemolyticus*	This study
pWP_005530005.1	pBAD33.1^{F} plasmid containing CDS of WP_005530005.1 from *V. campbellii* ATCC 25920 in frame with the C-terminal FLAG tag of the plasmid	Used for the arabinose-inducible expression of proteins in *V. parahaemolyticus*	This study
pWP_005530005.1$^{65-338}$	pBAD33.1^{F} plasmid containing CDS of WP_005530005.1$^{65-338}$ (truncation of the first 64 amino acids of	Used for the arabinose-inducible expression of proteins in *V. parahaemolyticus*	This study

	WP_005530005.1) in frame with the C-terminal FLAG tag of the plasmid		
pWP_038863399.1	pBAD33.1^F plasmid containing CDS of WP_038863399.1 from *V. campbellii* ATCC 25920 in frame with the C-terminal FLAG tag of the plasmid	Used for the arabinose-inducible expression of proteins in *V. parahaemolyticus*	This study
pWP_038863399.1$^{66-526}$	pBAD33.1^F plasmid containing CDS of WP_038863399.1$^{66-526}$ (truncation of the first 65 amino acids of WP_038863399.1) in frame with the C-terminal FLAG tag of the plasmid	Used for the arabinose-inducible expression of proteins in *V. parahaemolyticus*	This study
pEffector	pBAD33.1^F plasmid containing CDS of WP_005534959.1 from *V. campbellii* ATCC 25920 in frame with the C-terminal FLAG tag of the plasmid	Used for the arabinose-inducible expression of proteins in *E. coli* and *V. parahaemolyticus*	This study
pEffector+Immunity	pBAD33.1^F plasmid containing CDS of WP_005534959.1 and WP_005534960.1 from *V. campbellii* ATCC 25920 in frame with the C-terminal FLAG tag of the plasmid	Used for the arabinose-inducible expression of proteins in *E. coli* and *V. parahaemolyticus*	This study
pImmunity	pBAD33.1^F plasmid containing CDS of WP_005534960.1 in frame with the C-terminal FLAG tag of the plasmid	Used for the arabinose-inducible expression of proteins in *E. coli* and *V. parahaemolyticus*	This study
pRIX	pBAD33.1^F plasmid containing CDS of WP_157622110.1 from *V. coralliilyticus*	Used for the arabinose-inducible expression of proteins in *V.*	This study

	ATCC BAA-450 in frame with the C-terminal FLAG tag of the plasmid	*coralliilyticus* and *V. parahaemolyticus*	
pRte1	pBAD33.1^F plasmid containing CDS of WP_006958655.1 from *V. coralliilyticus* ATCC BAA-450 in frame with the C-terminal FLAG tag of the plasmid	Used for the arabinose-inducible expression of proteins in *E. coli*	This study
pRte1^{H94A}	pBAD33.1^F plasmid containing CDS of WP_006958655.1 from *V. coralliilyticus* ATCC BAA-450 with a substitution of histidine 94 for alanine, in frame with the C-terminal FLAG tag of the plasmid	Used for the arabinose-inducible expression of proteins in *E. coli* and the secretion assay in *V. parahaemolyticus*	This study
pRte1^{S106A}	pBAD33.1^F plasmid containing CDS of WP_006958655.1 from *V. coralliilyticus* ATCC BAA-450 with a substitution of serine 106 with alanine, in frame with the C-terminal FLAG tag of the plasmid	Used for the arabinose-inducible expression of proteins in *E. coli*	This study
pRIX+Rtei1	pBAD33.1^F plasmid containing CDS of WP_157622110.1, WP_006958655.1, and WP_050778602.1 from *V. coralliilyticus* ATCC BAA-450 in frame with the C-terminal FLAG tag of the plasmid	Used for the arabinose-inducible expression of proteins in *V. parahaemolyticus*	This study
pRIX$^{1-69}$+Rtei1	pRIX+Rtei1 in which the codon for amino acid 70 of WP_157622110.1	Used for the arabinose-inducible expression of proteins in *V. parahaemolyticus*	This study

	was replaced by a stop codon		
pRIX$^{65-134}$+Rtei1	pBAD33.1^{F} plasmid containing CDS of WP_157622110.1$^{65-134}$ (truncation of the first 64 amino acids of WP_157622110.1) WP_006958655.1, and WP_050778602.1 in frame with the C-terminal FLAG tag of the plasmid	Used for the arabinose-inducible expression of proteins in *V. parahaemolyticus*	This study
pRIX+Rte1^{H94A}	pBAD33.1^{F} plasmid containing CDS of WP_157622110.1 and WP_006958655.1 with a H94A mutation from *V. coralliilyticus* ATCC BAA-450 in frame with the C-terminal FLAG tag of the plasmid	Used for the arabinose-inducible expression of proteins in *V. parahaemolyticus*	This study
pRtei1	pBAD33.1^{F} plasmid containing CDS of WP_006958655.1 and WP_050778602.1 from *V. coralliilyticus* ATCC BAA-450 in frame with the C-terminal FLAG tag of the plasmid	Used for the arabinose-inducible expression of proteins in *V. parahaemolyticus*	This study
pGML10	pGML10 *E. coli-S. cerevisiae* shuttle vector, GAL1-10 promoter with a Myc tag at the 3' end of the MCS	Used for the galactose-inducible expression of proteins in *S. cerevisiae*	Riken
pGML10:eGFP	pGML10 containing the CDS of enhanced GFP in the EcoRI site of the MCS, in frame with the C-terminal Myc tag of the plasmid	Used for the galactose-inducible expression of eGFP in *S. cerevisiae*	This study

pGML10:WP_005536620.1	pGML10 containing the CDS of WP_005536620.1 in frame with the C-terminal Myc tag of the plasmid	Used for the galactose-inducible expression of proteins in *S. cerevisiae*	This study
pGML10:WP_005530005.1	pGML10 containing the CDS of WP_005530005.1 in frame with the C-terminal Myc tag of the plasmid	Used for the galactose-inducible expression of proteins in *S. cerevisiae*	This study
pGML10:WP_038863399.1	pGML10 containing the CDS of WP_038863399.1 in frame with the C-terminal Myc tag of the plasmid	Used for the galactose-inducible expression of proteins in *S. cerevisiae*	This study

Table S3. Primer pairs used in this study to construct plasmids.

Primer name	Sequence (5'->3') [a]	Comments
WP_050778602_pBAD^{M}_F	cgtcgtcatccttgtaatcCGTTCTTTCTTCTATAAACTC	Used to amplify the WP_050778602.1 CDS to construct pRti1^{M}
WP_050778602_pBAD^{M}_R	ttttgttcgggcccaagcttCGTTCTTTCTTCTATAAACTC	
Tme1$^{61-310}$_PER_F	cccagccggcgatggccATCTCTCAAGGTTTAAAAAC	Used to amplify the CDS of Tme1 amino acids 61-310 to construct pTme1$^{61-310/peri}$
Tme1_PER_R	ttttgttcgggcccaagcttTATCGCACTTTCTAACCTTTCAACTATCGTTTGAG	
Tme1_PER_F	cccagccggcgatggccATGAGATATGAATCCGCTCCTGTAGCCCC	Used to amplify the CDS of Tme1 amino acids 1-225 to constructpTme1$^{1-225/peri}$
Tme1$^{1-225}$_PER_R	ttttgttcgggcccaagcttCGCCGATACAACAAACGTTAAAACTCC	
Tme1_BAD_F	ctttaagaaggagatatacatATGAGATATGAATCCGCTCCTGTAGCCCC	Used to amplify the CDS of Tme1 to construct pTme1^{M}
Tme1_Myc_R	ttttgttcgggcccaagcttTATCGCACTTTCTAACCTTTC	
Tme1$^{61-310}$_BAD_F	ctttaagaaggagatatacatATGATCTCTCAAGGTTTAAAAAACGCC	Used to amplify the CDS of amino acids 61-310 of Tme1 together with primer Tme1_Myc_R to construct pTme1$^{61-310M}$
Tme1$^{1-60}$_linker_R	accaccagcagcagcTTGACCTAATCCAGC	Used to amplify the CDS of Tme1 amino acids 1-60 together

		with primer Tme1_BAD_F to construct pTme1$^{1-60}$-sfGFP
sfGFP_linker_F	gctgctgctggtggtggtATGGTGAGCAAGGGCGAGGAG	Used to amplify the CDS of sfGFP together with a linker sequence to construct pTme1$^{1-60}$-sfGFP
sfGFP_BAD_R	cgtcgtcatccttgtaatcCTTGTACAGCTCGTCCATGCCG	
WP_005536620.1_BAD_F	ctttaagaaggagatatacatATGAGATATGAATCCGCTCCTGTAGC	Used to amplify the CDS of WP_005536620.1 to construct pWP_005536620.1
WP_005536620.1_BAD_R	cgtcgtcatccttgtaatcGAAGCACTTCATGCTACTTTC	
WP_005536620.1$^{64-241}$_BAD_F	ctttaagaaggagatatacatATGGTAACAGCTAAGCTCCACCC	Used to amplify the CDS of WP_005536620.1 CDS for amino acids 64-241 together with primer WP_005536620.1_BAD_R to construct pWP_005536620.1$^{64-241}$
WP_005530005.1_BAD_F	ctttaagaaggagatatacatATGCGATATCAATCAGCGCC	Used to amplify the CDS of WP_005530005.1 to construct pWP_005530005.1
WP_005530005.1_BAD_R	cgtcgtcatccttgtaatcGCGCTTTATCGCGATGGTTC	
WP_005530005.1$^{65-338}$_BAD_F	ctttaagaaggagatatacatATGGCCGCGACAGAACAGAAAAACCATATTCAC	Used to amplify the CDS of WP_005530005.1 CDS for amino acids 65-338 together with primer WP_005530005.1_BAD_R to construct pWP_005530005.1$^{65-338}$
WP_038863399.1_BAD_F	ctttaagaaggagatatacatATGCCTAATCTTGATGAAC	Used to amplify the CDS of WP_038863399.1 to construct pWP_038863399.1
WP_038863399.1_BAD_R	cgtcgtcatccttgtaatcACCTCGTTTTTGAGAGCTATTAG	
WP_038863399.1$^{66-526}$_BAD_F	ctttaagaaggagatatacatATGCGTGAGCGAGCGGCTCAG	Used to amplify the CDS of WP_038863399.1 CDS for amino acids 66-526 together with primer

		WP_038863399.1_BAD_R to construct pWP_038863399.1$^{66-526}$
Effector_BAD_F	ctttaagaaggagatatacatATGTCGAATAGTTCTGATAATAATAAACTGTCAGC	Used to amplify the CDS of WP_005534959.1 to construct pEffector
Effector_BAD_R	cgtcgtcatccttgtaatcTTTAATTACTTTTCCTGAAC	
Immunity_BAD_F	ctttaagaaggagatatacatATGTCGAATAGTTCTGATAATAATAAACTGTCAGC	Used to amplify the CDS of WP_005534960.1 to construct pImmunity. Effector_BAD_F was used together with Immunity_BAD_R to amplify the CDS of WP_005534959.1 and WP_005534960.1 together to construct pEffector+Immunity
Immunity_BAD_R	cgtcgtcatccttgtaatcTGACTGTGAAGCTCTCTC	
RIX_BAD_F	ctttaagaaggagatatacatATGGTGACGATTTACAATAAAAAC	Used to amplify inserts containing the CDS of WP_157622110.1 to construct pRIX, pRIX+Rtei1, pRIX$^{1-69}$+Rtei1, and pRIX+Rte1^{H94A}
RIX_BAD_R	cgtcgtcatccttgtaatcGTCATGCCACCTCTCCAATG	Used to amplify the CDS of WP_157622110.1 to construct pRIX
Rte1_BAD_F	cccagccggcgatggccATGACTGATACGCTATTCAATACAACTATGG	Used to amplify inserts containing the CDS of WP_006958655.1 to construct pRte1, pRte1^{H94A}, pRte1^{S106A}, and pRtei1
Rte1_BAD_R	cgtcgtcatccttgtaatcGTTTTCATAAATAAAATCCGC	Used to amplify inserts containing the CDS of WP_006958655.1 to construct pRte1, pRte1^{H94A}, and pRte1^{S106A}
Rte1_H94A_F_KZ	CAAAAGCAAGTGTTGCTGAGGCTGTCATG	Used to amplify a H94A mutated

Rte1_H94A_R_KZ	CATGACAGCCTCAGCAACACTTGCTTTTG	version of WP_006958655.1
Rte1_S106A_F_KZ	CACTAAATCTCGGTTCGTTgctACGAGCTCAACC	Used to amplify a S106A mutated version of WP_006958655.1
Rte1_S106A_R_KZ	GGTTGAGCTCGTAGCAAagcACCGAGATTTAGTG	
Rti1_BAD_R	cgtcgtcatccttgtaatcCGTTCTTTCTTCTATAAACTC	Used to amplify inserts containing the CDS of WP_050778602.1 to construct pRIX+Rtei1, pRtei1, pRIX$^{1-69}$+Rtei1, and pRIX$^{65-134}$+Rtei1
RIX^{70stop}_F	CGCGAGTATTGGCATCTAGAtaaGTGGATCAACAAATGAG	Used to amplify a mutated version of WP_157622110.1 with a stop codon replacing the codon for amino acid 70, to construct pRIX$^{1-69}$+Rtei1
RIX^{70stop}_R	CTCATTTGTTGATCCACttaTCTAGATGCCAATACTCGCG	
RIX$^{65-134}$_BAD_F	ctttaagaaggagatatacatATGGTATTGGCATCTAGAATG	Used to amplify a truncated version of WP_157622110.1 starting with the codon for amino acid 65, to construct pRIX$^{65-134}$+Rtei1
WP_005536620.1_pGML_F	accccggatcgaattgactctagaATGAGATATGAATCCGCTCCTGTAGC	Used to amplify the CDS of WP_005536620.1 to construct pGML10:WP_005536620.1
WP_005536620.1_pGML_R	tttctgttcagaatt GTCATGCCACCTCTCCAATG	
WP_005530005.1_pGML_F	accccggatcgaattgactctagaATGCGATATCAATCAGC	Used to amplify the CDS of WP_005530005.1 to construct pGML10:WP_005530005.1
WP_005530005.1_pGML_R	tttctgttcagaattGCGCTTTATCGCGATGGTTC	
WP_038863399.1_pGML_F	accccggatcgaattgactctagaATGCCTAATCTTGATGAACTAAAAAG	Used to amplify the CDS of WP_038863399.1 to construct pGML10:WP_038863399.1
WP_038863399.1_pGML_R	tttctgttcagaattACCTCGTTTTTGAGAGCTATTAGATGACG	
pGML10_Gib_F	aattctgaacagaaactgatttccgaagaggatctg	Used to amplify the pGML10 plasmid
pGML10_Gib_R	tctagagtcaattcgatccggggt	

		backbone for Gibson assembly
pBAD33.1F_Gib_F	GATTACAAGGATGACGACGataagtgaaagcttggctgttttggcgg	Used to amplify the pBAD33.1 plasmid backbone with a C-terminal FLAG tag for Gibson assembly
pBAD33.1F_Gib_R	catatgtatatctccttcttaaagttaaacaaaattatttctagag	
pBAD33.1M_Gib_F	ACAAAAACTCATCTCAGAAGAGGATCTGtgaaagcttggctgttttg	Used to amplify the pBAD33.1 plasmid backbone with a C-terminal Myc-His tag for Gibson assembly (together with primer pBAD33.1F_Gib_F)
pPER5_Gib_F	aagcttgggcccgaacaaaaac	Used to amplify the pPER5 plasmid backbone for Gibson assembly
pPER5_Gib_R	ggccatcgccggctgggcagcg	

[a] Uppercase letters correspond to gene sequences; lowercase letters correspond to plasmid sequences

References

1. McCarter, L. L. OpaR, a homolog of Vibrio harveyi LuxR, controls opacity of Vibrio parahaemolyticus. *J Bacteriol* **180**, (1998).

2. Fridman, C. M., Keppel, K., Gerlic, M., Bosis, E. & Salomon, D. A comparative genomics methodology reveals a widespread family of membrane-disrupting T6SS effectors. *Nat Commun* **11**, 1085 (2020).

3. Jana, B., Keppel, K., Fridman, C. M., Bosis, E. & Salomon, D. Multiple T6SSs, Mobile Auxiliary Modules, and Effectors Revealed in a Systematic Analysis of the Vibrio parahaemolyticus Pan-Genome. *mSystems* (2022) doi:10.1128/MSYSTEMS.00723-22.

4. Salomon, D., Gonzalez, H., Updegraff, B. L. & Orth, K. Vibrio parahaemolyticus Type VI secretion system 1 Is activated in marine conditions to target bacteria, and is differentially regulated from system 2. *PLoS One* **8**, e61086 (2013).

5. Dar, Y., Salomon, D. & Bosis, E. The antibacterial and anti-eukaryotic Type VI secretion system MIX-effector repertoire in Vibrionaceae. *Mar Drugs* **16**, 433 (2018).

6. Dar, Y., Jana, B., Bosis, E. & Salomon, D. A binary effector module secreted by a type VI secretion system. *EMBO Rep* **23**, e53981 (2022).
