# Supplementary material for: The RIX domain defines a class of polymorphic T6SS effectors and secreted adaptors
Source: Nat Commun. 2023 Aug 17;14:4983. doi: 10.1038/s41467-023-40659-2 (PMC10435454; doi:10.1038/s41467-023-40659-2)
Supplement: Supplementary file 3 — Description of Additional Supplementary Files [file 41467_2023_40659_MOESM3_ESM.pdf]

Description of Additional Supplementary Files:

Supplementary Data 1: RIX domain-containing proteins identified in this study.

Supplementary Data 2: The presence of T6SS in RIX domain-encoding bacterial genomes.

Supplementary Data 3: AlphaFold2-generated PDB files used in this study.
