# Supplementary material for: The RIX domain defines a class of polymorphic T6SS effectors and secreted adaptors
Source: Nat Commun. 2023 Aug 17;14:4983. doi: 10.1038/s41467-023-40659-2 (PMC10435454; doi:10.1038/s41467-023-40659-2)
Supplement: Supplementary file 7 — Reporting Summary [file 41467_2023_40659_MOESM7_ESM.pdf]

nature portfolio

Corresponding author(s): Dor Salomon and Eran Bosis

Last updated by author(s): 26/07/2023

Reporting Summary

Nature Portfolio wishes to improve the reproducibility of the work that we publish. This form provides structure for consistency and transparency in reporting. For further information on Nature Portfolio policies, see our Editorial Policies and the Editorial Policy Checklist.

Statistics

For all statistical analyses, confirm that the following items are present in the figure legend, table legend, main text, or Methods section.

n/a	Confirmed	
☐	☒	The exact sample size (*n*) for each experimental group/condition, given as a discrete number and unit of measurement
☐	☒	A statement on whether measurements were taken from distinct samples or whether the same sample was measured repeatedly
☐	☒	The statistical test(s) used AND whether they are one- or two-sided<br>*Only common tests should be described solely by name; describe more complex techniques in the Methods section.*
☒	☐	A description of all covariates tested
☒	☐	A description of any assumptions or corrections, such as tests of normality and adjustment for multiple comparisons
☐	☒	A full description of the statistical parameters including central tendency (e.g. means) or other basic estimates (e.g. regression coefficient) AND variation (e.g. standard deviation) or associated estimates of uncertainty (e.g. confidence intervals)
☐	☒	For null hypothesis testing, the test statistic (e.g. *F*, *t*, *r*) with confidence intervals, effect sizes, degrees of freedom and *P* value noted<br>*Give P values as exact values whenever suitable.*
☒	☐	For Bayesian analysis, information on the choice of priors and Markov chain Monte Carlo settings
☒	☐	For hierarchical and complex designs, identification of the appropriate level for tests and full reporting of outcomes
☒	☐	Estimates of effect sizes (e.g. Cohen's *d*, Pearson's *r*), indicating how they were calculated

Our web collection on statistics for biologists contains articles on many of the points above.

Software and code

Policy information about availability of computer code

Data collection	NCBI Blast+ 2.10.0 suite
Data analysis	CLANS (29.05.2012), GraphPad PRISM 9, Excel 2019, iTOL v6, AlphaFold2, MAFFT v7.505, Clustal Omega v1.2.4, WebLogo, WebLogo 3, NCBI's CDD v3.19, ChimeraX v1.4, HHpred

For manuscripts utilizing custom algorithms or software that are central to the research but not yet described in published literature, software must be made available to editors and reviewers. We strongly encourage code deposition in a community repository (e.g. GitHub). See the Nature Portfolio guidelines for submitting code & software for further information.

Data

Policy information about availability of data

All manuscripts must include a data availability statement. This statement should provide the following information, where applicable:

- Accession codes, unique identifiers, or web links for publicly available datasets
- A description of any restrictions on data availability
- For clinical datasets or third party data, please ensure that the statement adheres to our policy

The experimental and computational data that support the findings of this research are available in this article and its supplementary information files. Source data are provided with this paper.

Research involving human participants, their data, or biological material

Policy information about studies with human participants or human data. See also policy information about sex, gender (identity/presentation), and sexual orientation and race, ethnicity and racism.

Reporting on sex and gender	NA
Reporting on race, ethnicity, or other socially relevant groupings	NA
Population characteristics	NA
Recruitment	NA
Ethics oversight	NA

Note that full information on the approval of the study protocol must also be provided in the manuscript.

Field-specific reporting

Please select the one below that is the best fit for your research. If you are not sure, read the appropriate sections before making your selection.

[x] Life sciences [ ] Behavioural & social sciences [ ] Ecological, evolutionary & environmental sciences

For a reference copy of the document with all sections, see nature.com/documents/nr-reporting-summary-flat.pdf

Life sciences study design

All studies must disclose on these points even when the disclosure is negative.

Sample size	Sample sizes of 3 or 4 samples were used in growth assays and competition assays. The were chosen since they are the minimal sample size required for statistical analyses, and because they are used in the majority of similar manuscripts in the field.
Data exclusions	No data were excluded from analyses.
Replication	All experiments were performed at least twice with similar results
Randomization	Experiments were repeated independently on different dates, yielding similar results.
Blinding	Blinding was not performed since the assays described in the manuscript are such that are setup, performed, and analyzed by the same individual.

Reporting for specific materials, systems and methods

We require information from authors about some types of materials, experimental systems and methods used in many studies. Here, indicate whether each material, system or method listed is relevant to your study. If you are not sure if a list item applies to your research, read the appropriate section before selecting a response.

Materials & experimental systems

n/a	Involved in the study	
[ ]	[x]	Antibodies
[x]	[ ]	Eukaryotic cell lines
[x]	[ ]	Palaeontology and archaeology
[x]	[ ]	Animals and other organisms
[x]	[ ]	Clinical data
[x]	[ ]	Dual use research of concern
[x]	[ ]	Plants

Methods

n/a	Involved in the study	
[x]	[ ]	ChIP-seq
[x]	[ ]	Flow cytometry
[x]	[ ]	MRI-based neuroimaging

Antibodies

Antibodies used	1) α-FLAG M2 (Sigma-Aldrich, F1804)<br>2) α-Myc (Santa Cruz, 9E10, sc-40)<br>3) α-β-actin antibodies (Santa Cruz, sc-47778, C4)<br>4) Direct-BlotTM HRP anti-E. coli RNA polymerase Sigma 70 (mouse mAb #663205; BioLegend)

	5) Custom-made α-VgrG1 (Polyclonal antibodies were produced in-house with rabbits for the Vibrio parahaemolyticus VgrG1 (VP1394) peptide KDMSTKVLNNRYRDIGQDE and were affinity purified (GenScript))
Validation	1) α-FLAG M2 manufacturer website: https://www.sigmaaldrich.com/IL/en/product/sigma/f1804<br>2) α-c-Myc manufacturer website: https://www.scbt.com/scbt/product/c-myc-antibody-9e10<br>3) α-β-actin manufacturer website: https://www.scbt.com/p/beta-actin-antibody-c4<br>4) α-RNA polymerase sigma 70 manufacturer website: https://www.biolegend.com/en-us/products/direct-blot-hrp-anti-eme-coli-em-rna-sigma-70-antibody-13486<br>5) VgrG1 Antibody specificity was tested in-house using Vibrio wild-type or vgrG1-deletion strains. This antibody was also used in DOI: 10.1128/AEM.00737-17 and in DOI 10.15252/embr.201744226
