# Supplementary material for: The RIX domain defines a class of polymorphic T6SS effectors and secreted adaptors
Source: Nat Commun. 2023 Aug 17;14:4983. doi: 10.1038/s41467-023-40659-2 (PMC10435454; doi:10.1038/s41467-023-40659-2)
Supplement: Supplementary file 8 — Source Data [file 41467_2023_40659_MOESM8_ESM.zip › Source data - uncropped blots.pdf]

Uncropped blots for Fig. 1B

Media Cells

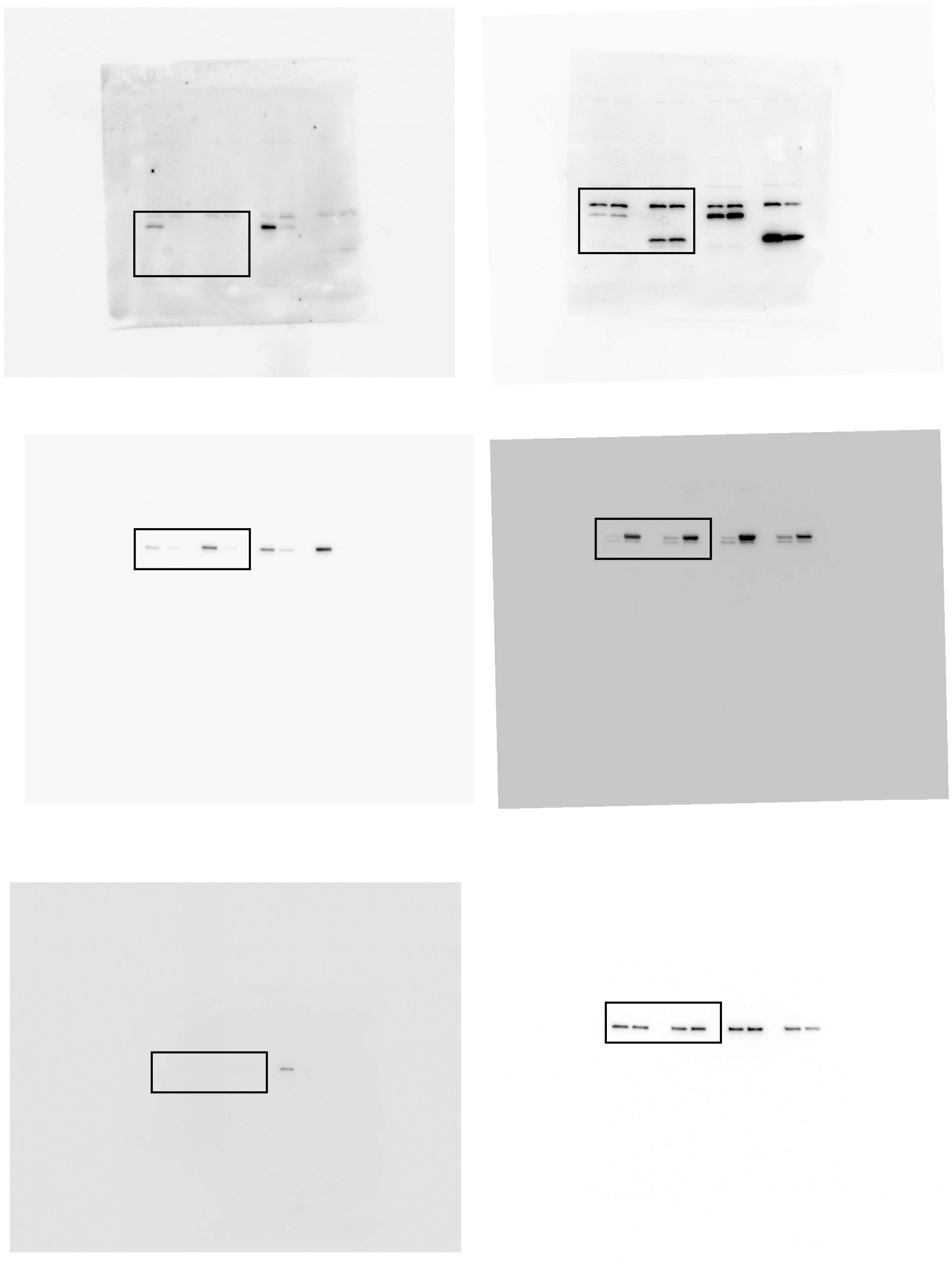

α–Myc

α–VgrG1

α–RNAp

Uncropped blots for Fig. 1C

Media | Cells

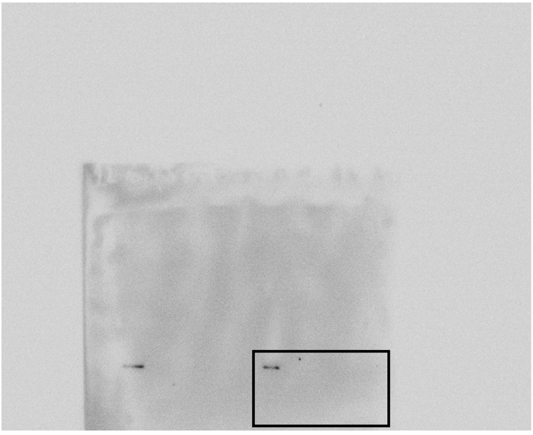

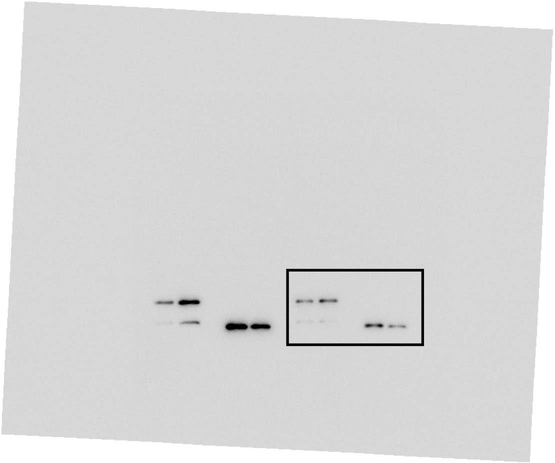

α–FLAG

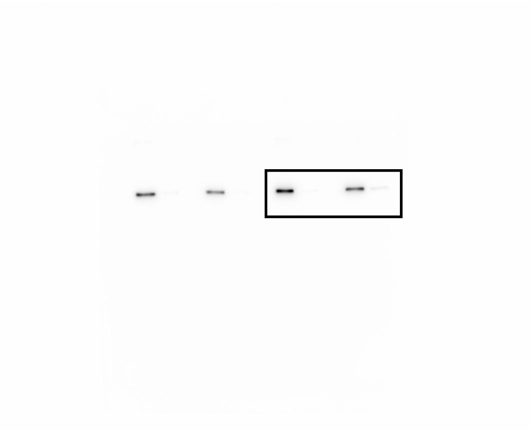

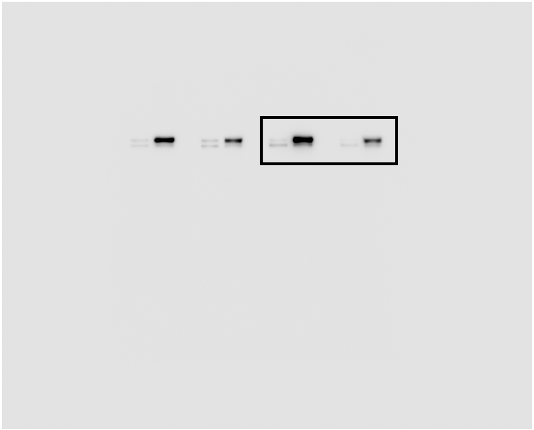

α–VgrG1

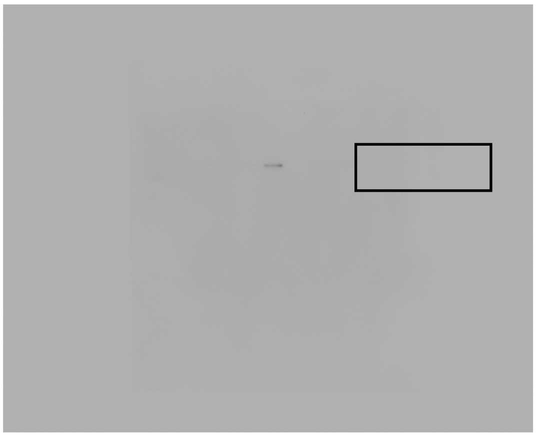

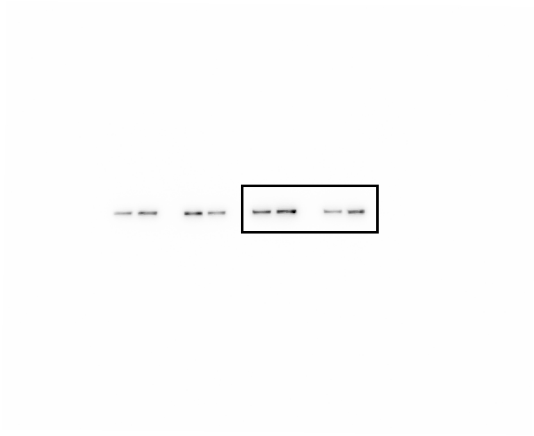

α–RNAp

Uncropped blots for Fig. 3B
Left panel (WP_005536620.1)

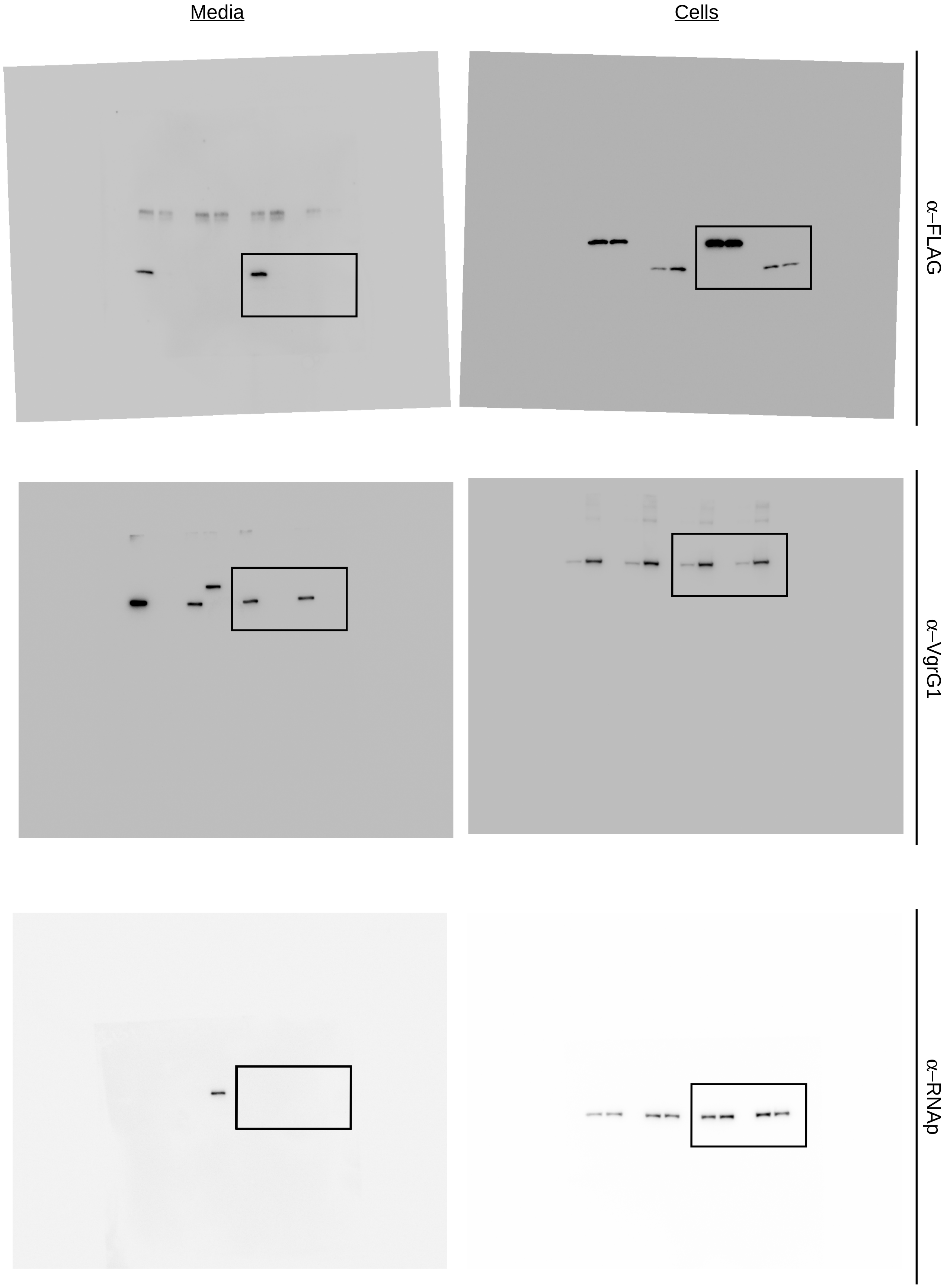

Uncropped blots for Fig. 3B
Middle panel (WP_005530005.1)

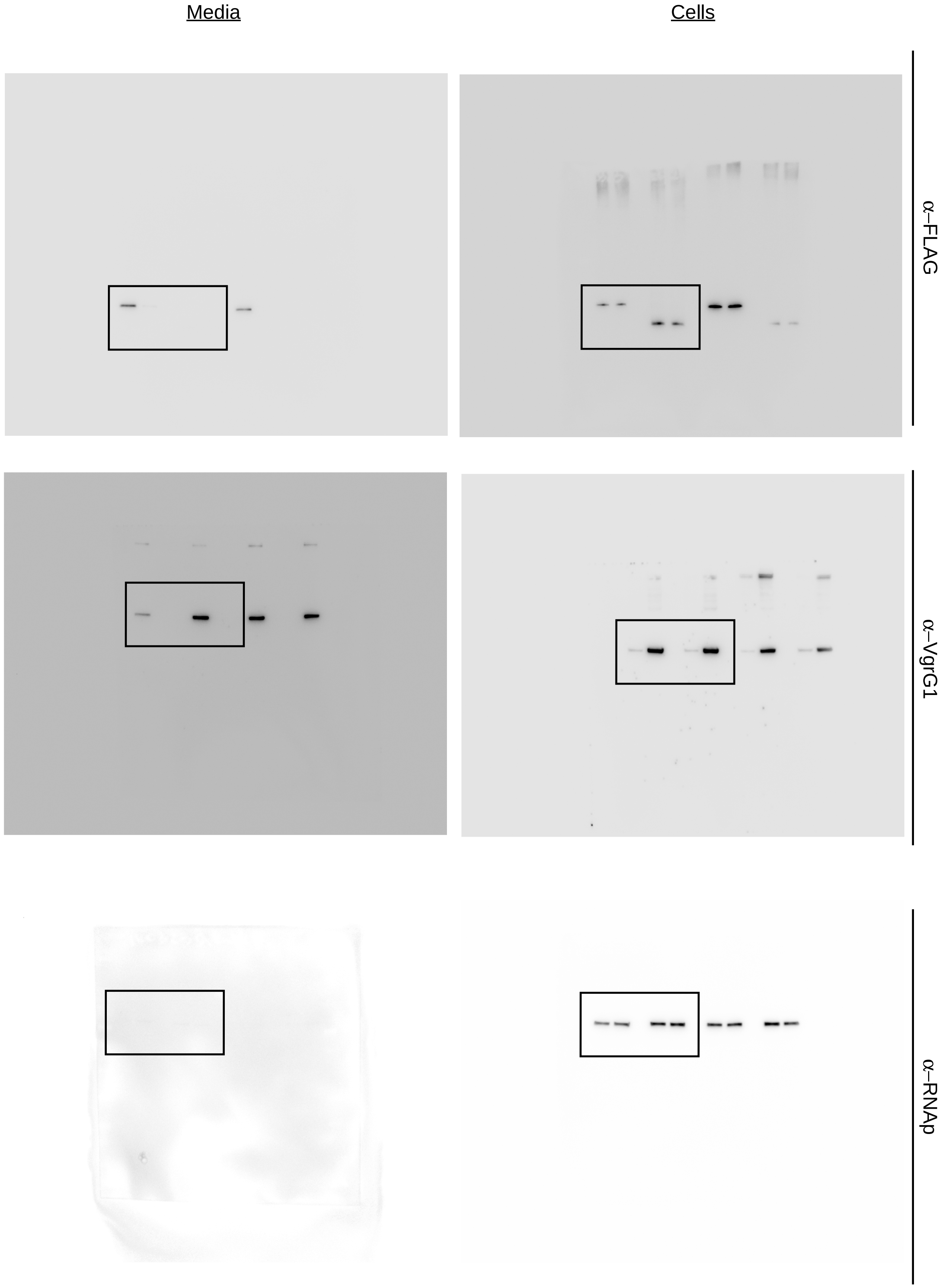

Uncropped blots for Fig. 3B
Right panel (WP_038863399.1)

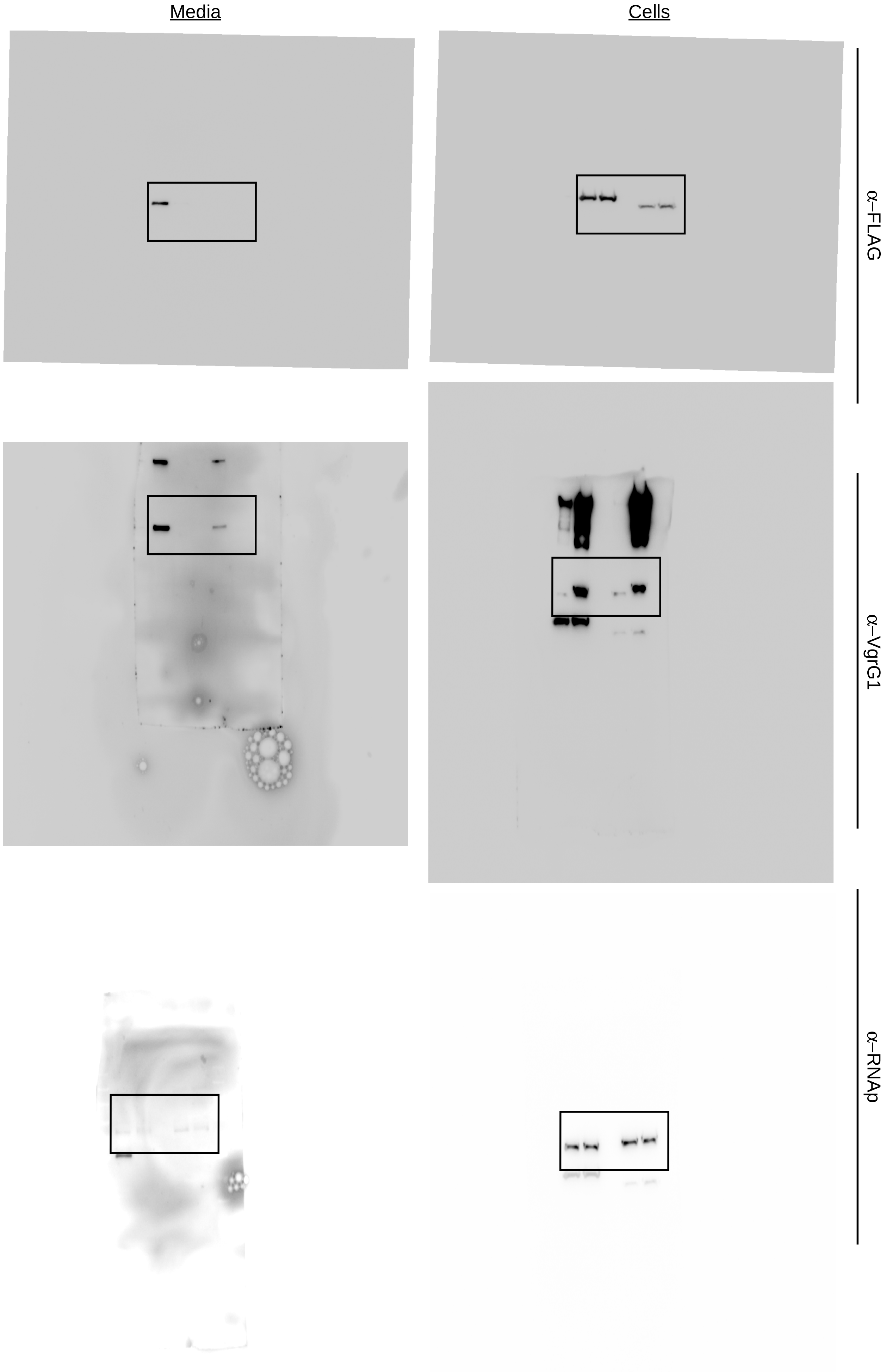

Uncropped blots for Fig. 4A

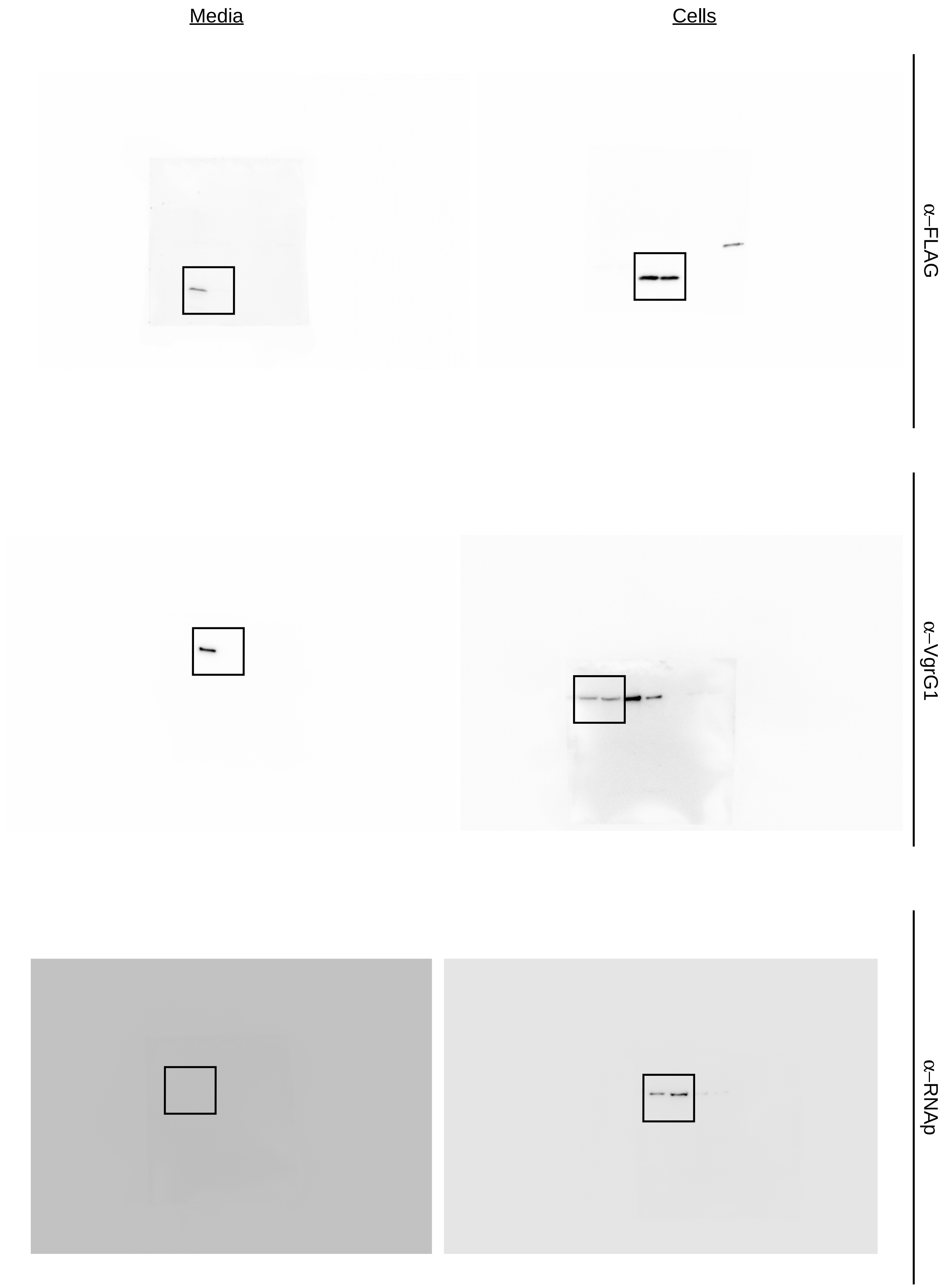

Uncropped blots for Fig. 4B

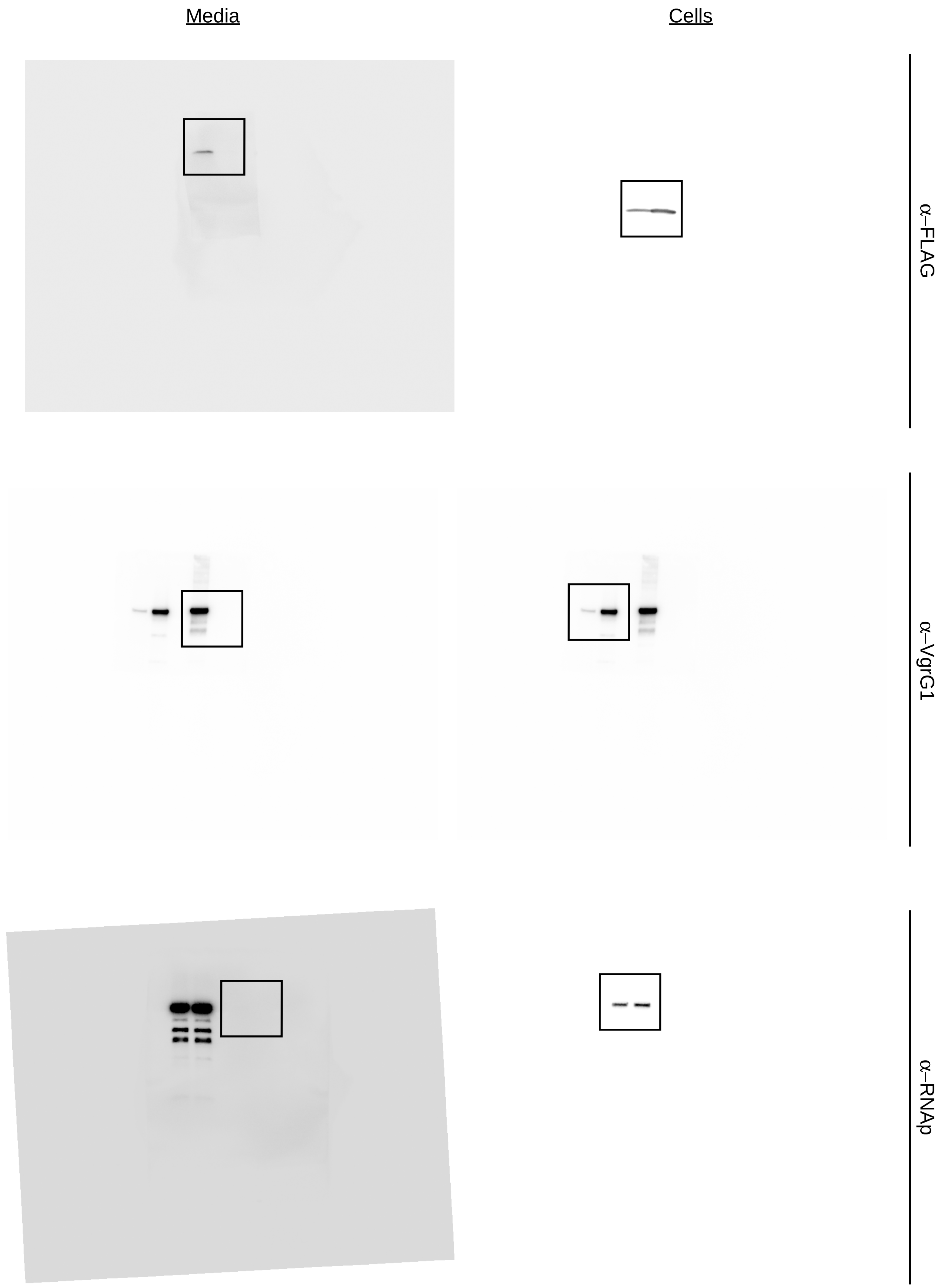

Uncropped blots for Fig. 5D

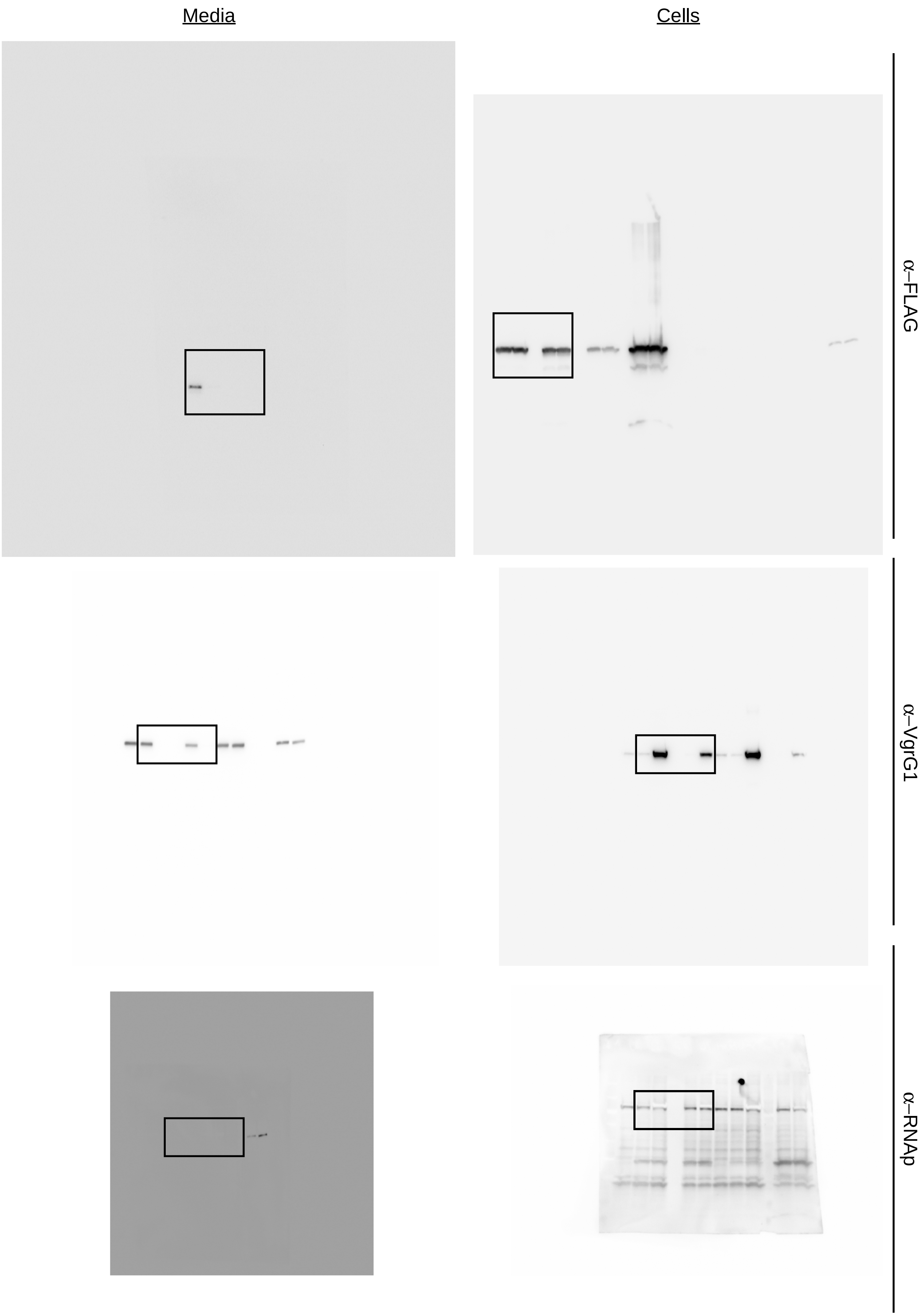

Uncropped blots for Fig. S1

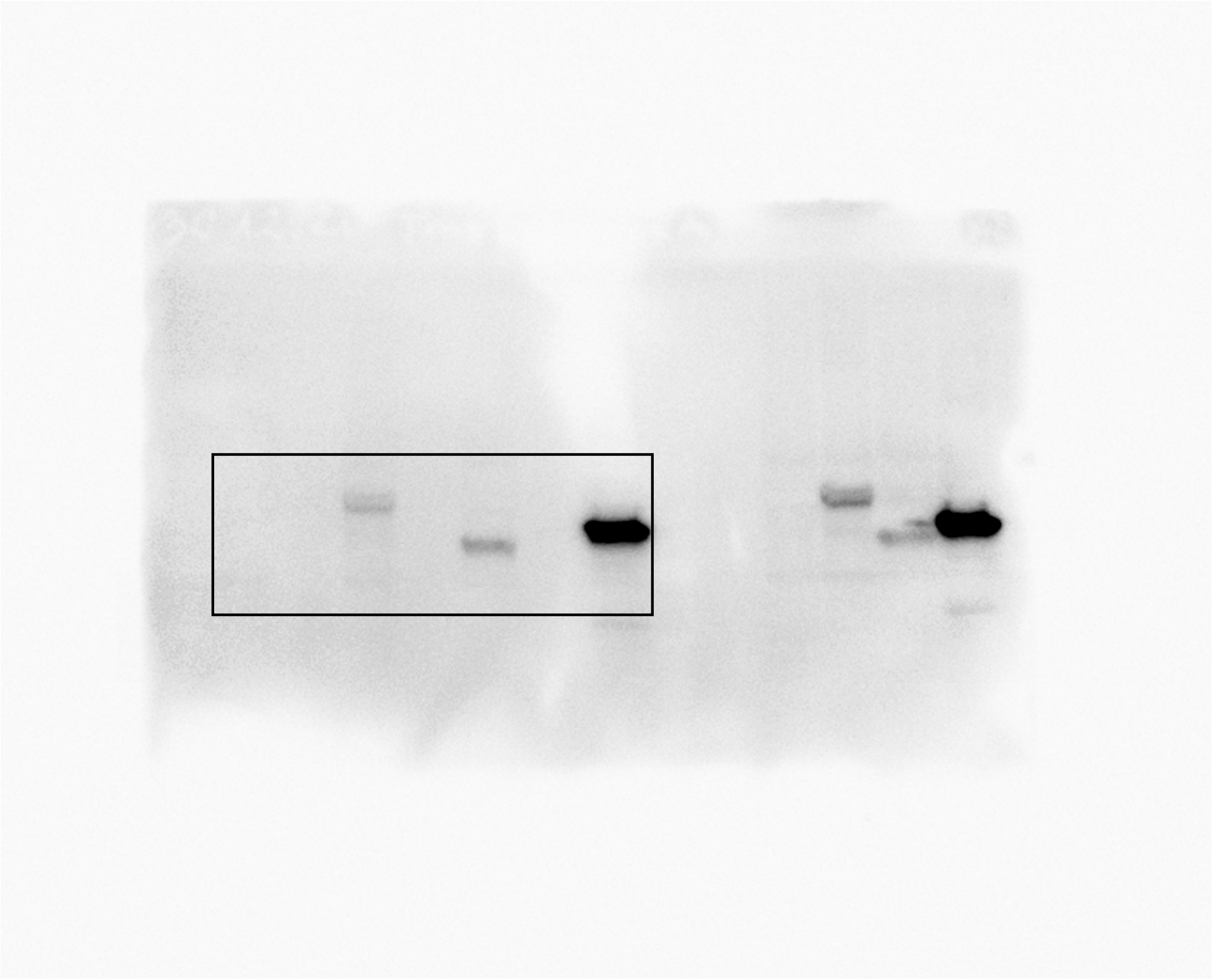

α–Myc

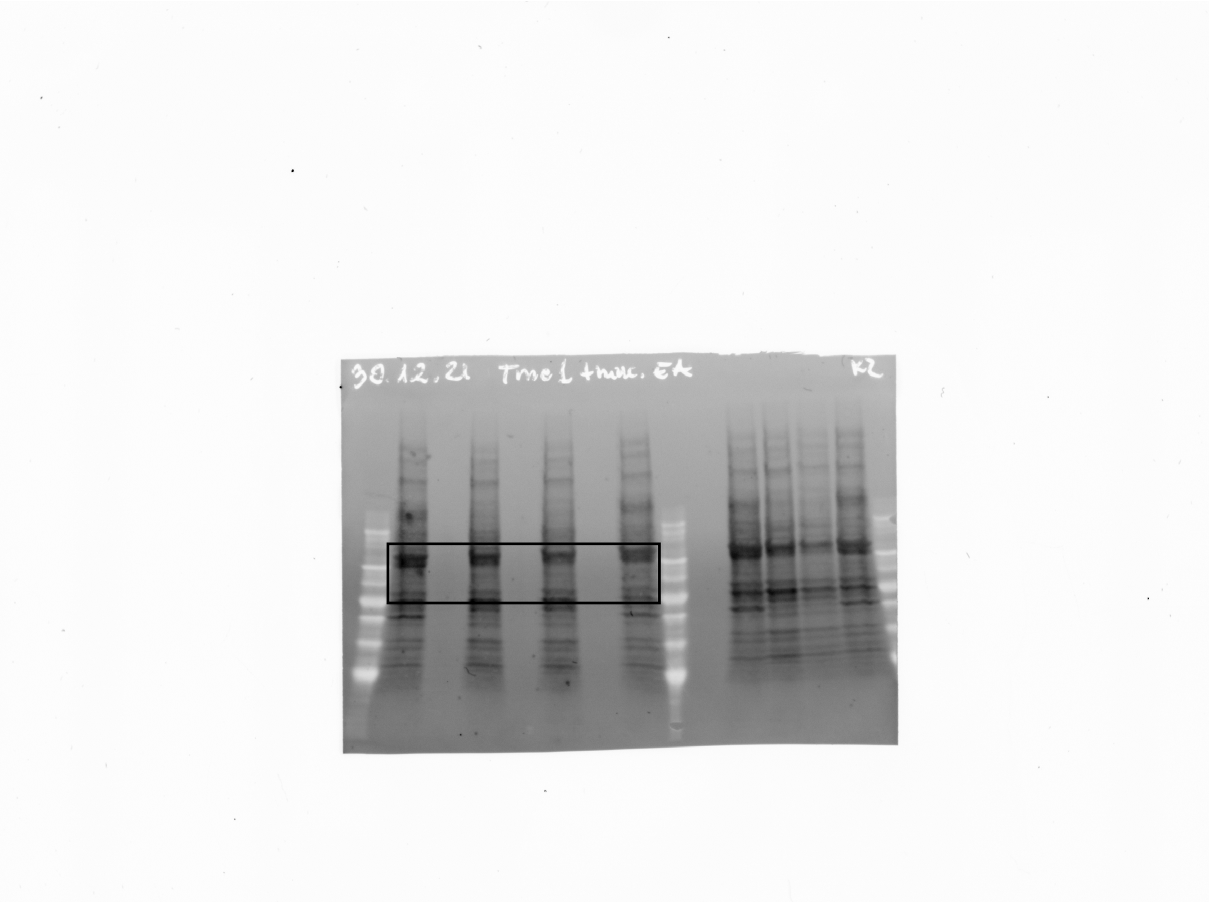

LC

Uncropped blots for Fig. S6A

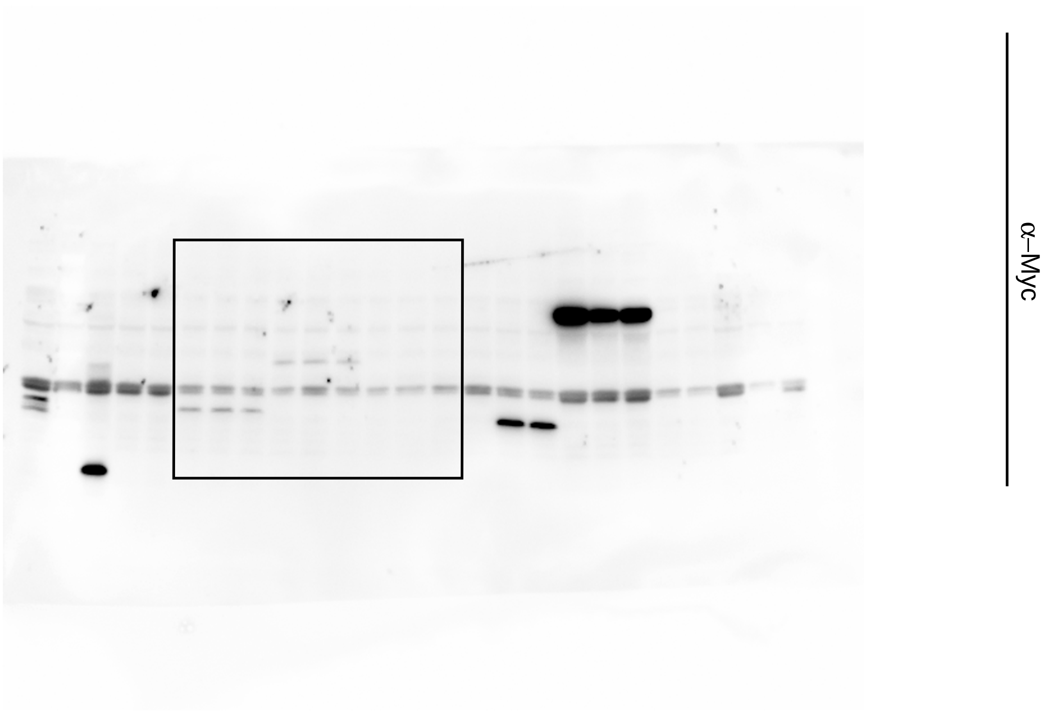

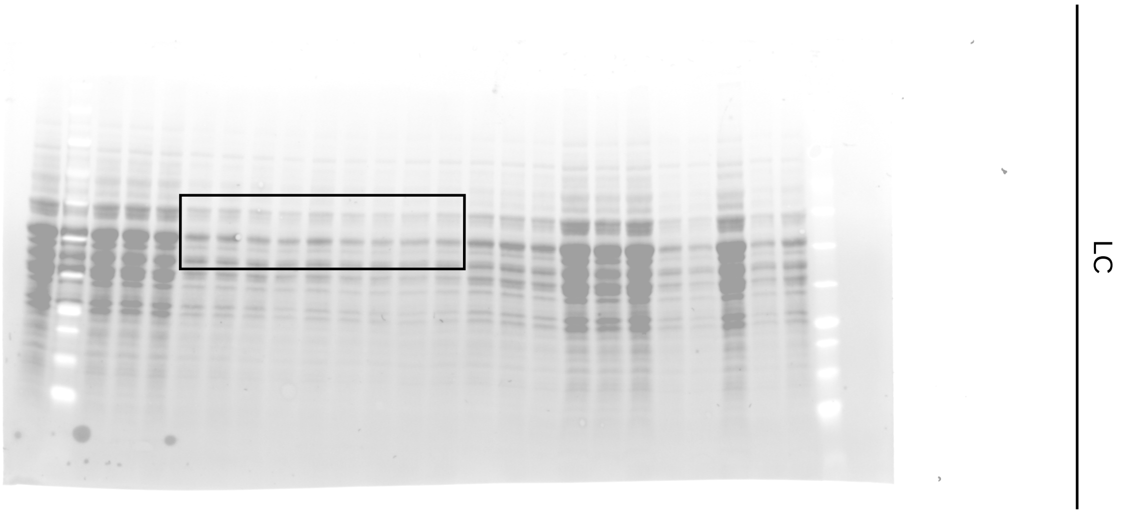

Uncropped blots for Fig. S6A

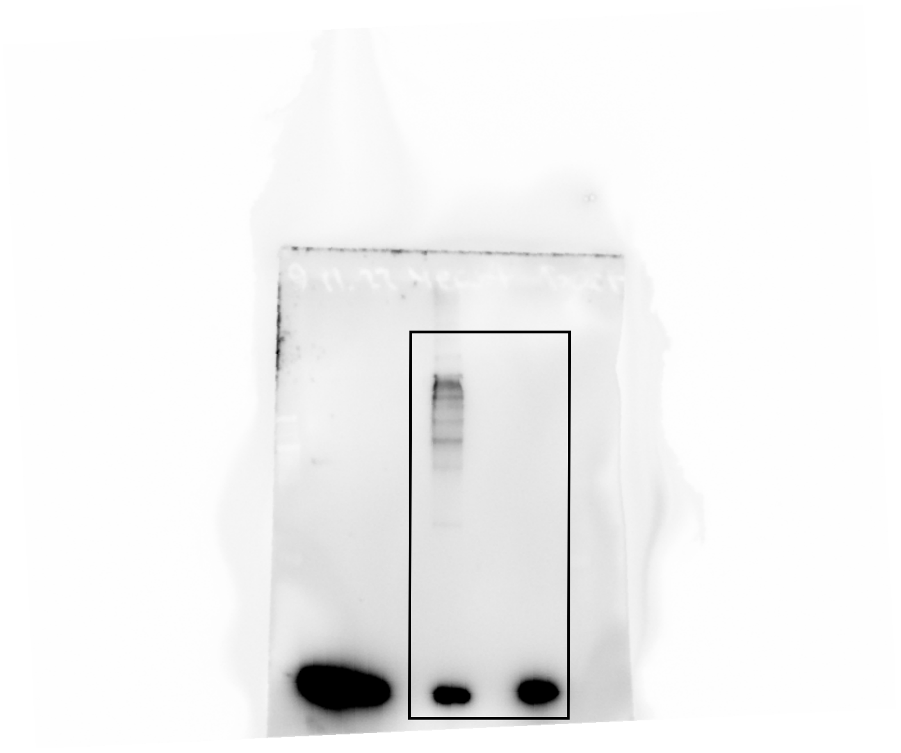

α–β-Actin

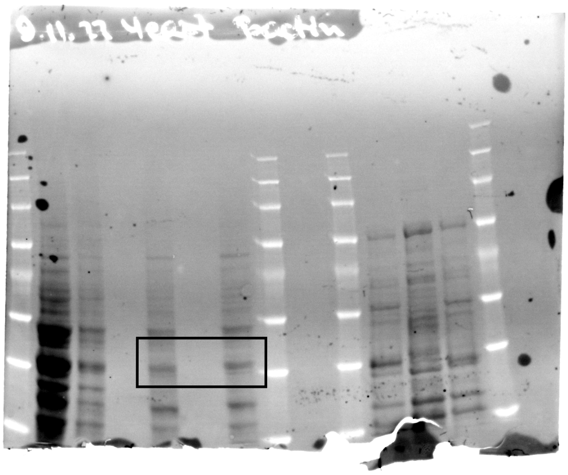

LC

Uncropped blots for Fig. S7A

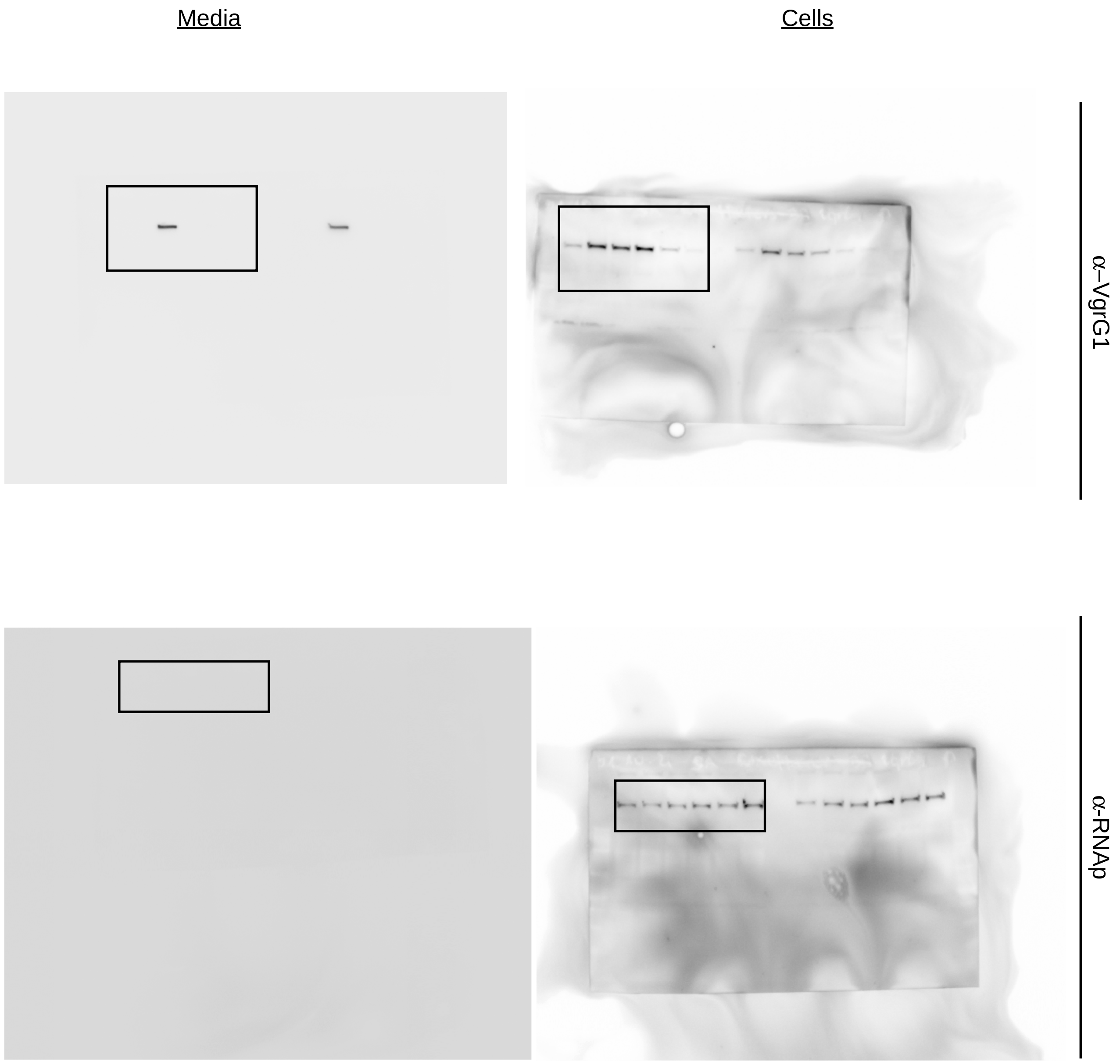

Uncropped blots for Fig. S8F

Left panels

Right panels

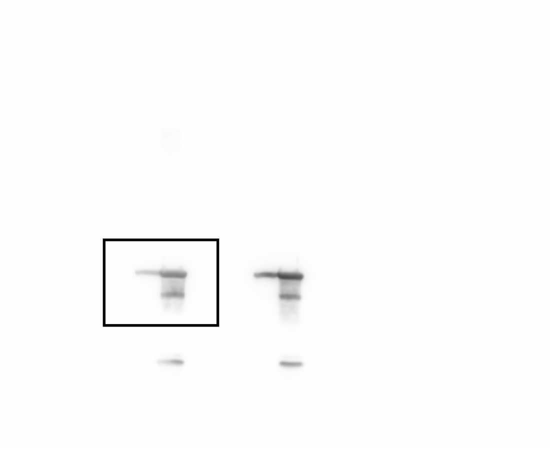

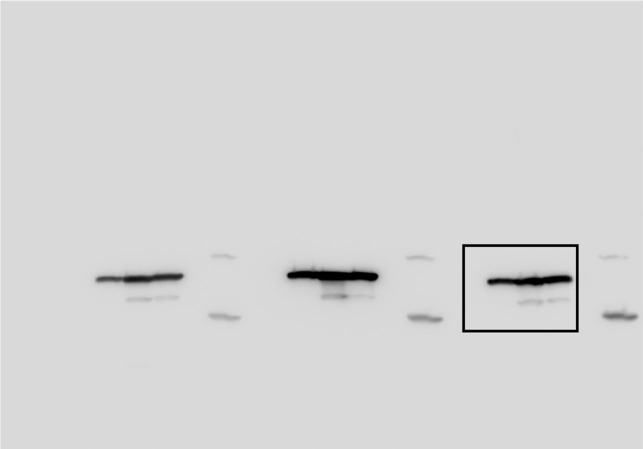

α–FLAG

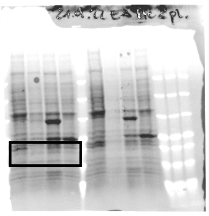

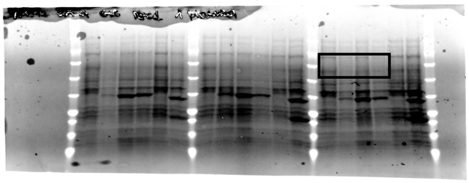

LC
